# Early-life gut microbiome maturity regulates blood–brain barrier and cognitive development

**DOI:** 10.1080/19490976.2025.2551879

**Published:** 2025-08-31

**Authors:** Zachary M. Zemmel, Xiaobing Fan, Yueyue Yu, Erica Markiewicz, Hsiu-Ming Tsai, Lei Lu, Jessica C. Little, Ramanujam Ramaswamy, Bree Andrews, Erika C. Claud, Jing Lu

**Affiliations:** a Princeton Neuroscience Institute, Princeton University, Princeton, NJ, USA; b University of Chicago Neuroscience Institute, Chicago, IL, USA; c Department of Radiology, University of Chicago, Chicago, IL, USA; d Center for the Science of Early Trajectories, University of Chicago, Chicago, IL, USA; e Department of Pediatrics, University of Chicago, Chicago, IL, USA; f Johnson & Johnson Innovative Medicine, San Diego, CA, USA; g Duchossois Family Institute, University of Chicago, Chicago, IL, USA; h Department of Pediatrics, Comer Children’s Hospital, Chicago, IL, USA; i Department of Pediatrics, Pritzker School of Medicine, Chicago, IL, USA

**Keywords:** blood–brain barrier, gut–brain axis, neurodevelopmental impairment, single-cell RNA sequencing, magnetic resonance imaging, shotgun metagenomics, metabolomics, host–microbe interactions

## Abstract

The gut microbiome is an emerging factor in the neurobiology of disease. Blood–brain barrier (BBB) integrity is essential for proper brain function. However, the role the initial microbiome plays in BBB and brain development is unclear. In this study, we colonized germ-free pregnant mice with human full-term- or preterm-infant-derived gut microbiota, thereby establishing these communities in the resulting offspring. We discovered that mice harboring a full-term-associated microbiome exhibited stronger memory and learning capabilities and dramatically decreased early-life BBB permeability when compared to those with a prematurity-associated microbiome. Whole-brain single-cell RNA sequencing revealed downregulation of synaptic signaling genes in BBB cell types of mice with the prematurity-associated microbiome, indicating that microbiome maturity influences BBB transcriptional programs that support cognitive development. Comprehensive metagenomics and metabolomics uncovered bacterial populations and genomic pathways corresponding with decreased levels of circulating long-chain acylcarnitines and lysophosphatidylcholines in mice with the full-term-associated microbiome. Our findings highlight the microbiome as a therapeutic target for improving long-term neurodevelopmental outcomes due to its effect on the early-life BBB.

## Introduction

The gut microbiome-brain axis is a complex, bidirectional communication network that plays a pivotal role in human health and development. This intricate network encompasses the central nervous system (CNS), the enteric nervous system, the neuroendocrine and neuroimmune systems, and the gut microbiota. The harmonious interplay between these components is crucial for maintaining both healthy gut physiology and host behavior and cognition. Therefore, perturbations in this balance can have substantial implications for gut physiology, gut-brain axis signaling, and proper CNS function.^1–4^ In particular, the gut microbiome is now an emerging factor in a variety of neurodegenerative diseases, including Parkinson’s Disease, Multiple Sclerosis, and Alzheimer’s Disease.^5–10^

Preterm infants are at risk for long-term cognitive impairment.^11,12^ Several studies have investigated the succession of the preterm infant microbiome and found that it follows a unique maturational cadence and differs from that of a full-term infant.^13–16^ The degree of immaturity of the preterm infant microbiome, defined by postmenstrual age, also predicts the neurodevelopmental trajectory in a humanized mouse model.^17^ However, the potential underlying mechanisms by which the preterm infant microbiome affects brain development remain underexplored. Identifying and characterizing the key biological interfaces mediating gut–brain interactions during early life would provide valuable insight into the pathogenesis of microbiome-associated neurodevelopmental deficits.

The blood–brain barrier (BBB) is a compelling candidate. The BBB is a specialized vascular interface that regulates molecular and cellular exchange between the bloodstream and the CNS and is composed of endothelial cells (ECs), mural cells, astrocytic end-feet, and the basement membrane. While the BBB is essential for maintaining brain homeostasis, BBB dysfunction is increasingly recognized as a key contributor to neurological disease, with vascular breakdown leading to neuroinflammation, impaired repair, and neuronal damage across a range of diseases.^18–20^ Novel single-cell transcriptomics studies demonstrate that BBB cell populations exhibit significant heterogeneity across brain regions, disease states, and ages, suggesting that targeted examination of these populations in development is necessary to explain possible long-term effects of early-life neurovascular dysfunction.^20–24^ Previous research has found that the gut microbiome may influence the BBB through microbial metabolites, which impact BBB permeability in comparisons between germ-free and specific-pathogen-free mice.^25,26^ Furthermore, recent work from our group suggests that probiotic interventions may help restore normal BBB development after disruption to the microbiome.^27^ More broadly, gut-derived metabolites and microbial byproducts modulate BBB function in multiple model systems.^28,29^ However, the extent to which differences between the preterm and full-term microbiome impact BBB-regulated neurodevelopment remains unknown.

We hypothesized that mice harboring a full-term infant microbiome would exhibit preserved cognitive function and intact BBB development compared to those with a preterm infant microbiome. To that end, we transplanted germ-free pregnant mice with human full-term- or preterm-infant-derived gut microbiota and conducted behavioral, radiological, and multi-omic profiling to evaluate offspring neurodevelopment. We found that mice colonized with full-term microbiota exhibited stronger cognitive function, lower early-life BBB permeability, intact expression of genes and proteins related to control of synaptic signaling in BBB-associated cell types, and lower levels of potentially harmful microbiota-dependent serum metabolites, particularly acylcarnitines and lysophosphatidylcholines. Our work demonstrates that a full-term microbiome supports brain development, likely by maturing the function of the developing BBB and BBB-derived regulation of synaptic signaling.

## Results

**Figure 1.**
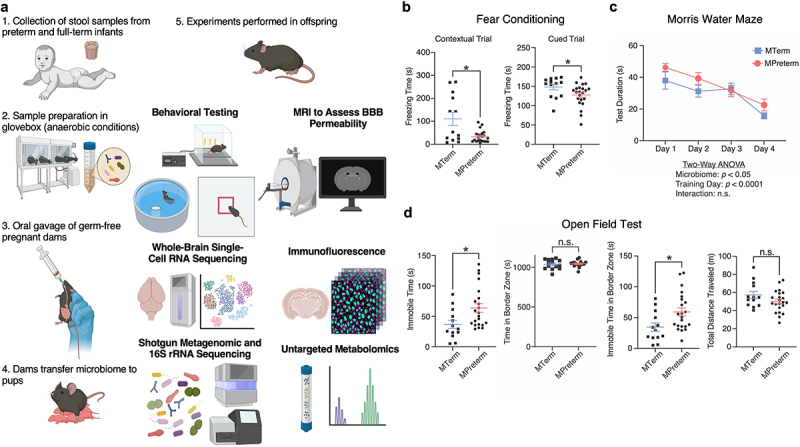
Compared to those colonized with the full-term infant microbiome, mice colonized with the preterm infant microbiome exhibit behavioral deficits. (a) Experimental schematic. Created in BioRender. Zemmel, Z. (2025) https://biorender.com/lnqm8du. (b–d) results of behavioral assays conducted at four weeks of age. Data presented as mean ± SEM. (b) Cued fear conditioning. MPreterm mice displayed associative learning deficits. Freezing times for MPreterm mice were significantly lower in contextual (mice returned to same environment) and cued (mice returned to different environment of the chamber and sound cue presented 24 h after the conditioning) fear conditioning tests. *p < 0.05, Welch’s *t*-test. (c) Morris Water Maze. MPreterm mice displayed spatial learning deficits. Data were plotted using test duration (latency to reach the escape platform, or when by 60 s a mouse has not reached the platform, the number was assigned as 60 s). Two-way ANOVA: main effect of training day: *p* < 0.0001, main effect of microbiome: *p* < 0.05, interaction: not significant (*p* = 0.539). (d) Open field test. MPreterm mice displayed anxiety-like behaviors. MPreterm mice spent significantly more immobile time overall and in the border zone. n.s. not significant, * *p* < 0.05, Welch’s *t*-test.

To investigate the impact of microbiome maturity on neurodevelopment, we established a gnotobiotic-humanized mouse model by colonizing germ-free pregnant C57BL/6J mice with human microbiota from either a full-term infant (gestational age (GA) = 39 weeks) (MTerm) or a preterm infant (GA = 24 weeks) (MPreterm). Following gavage of the pregnant dams with fecal preparations, pups were delivered naturally and acquired the transplanted microbiota starting at birth. This approach allowed us to directly compare the neurodevelopmental outcomes in offspring harboring gut microbiomes of either mature or immature human origin (Figure 1(a)).

We validated our microbiota transplantation approach by performing 16S ribosomal RNA sequencing (16S rRNA-seq) of fecal samples from the human donors and from MTerm and MPreterm mice at two weeks postnatal (*n*_*MTerm*_ = 8, *n*_*MPreterm*_ = 6), four weeks postnatal (*n*_*MTerm*_ = 12, *n*_*MPreterm*_ = 6), and dams (*n*_*MTerm*_ = 6, *n*_*MPreterm*_ = 6). Consistent with previous studies, the gut microbiome of the full-term infant donor had considerably lower *Proteobacteria*, higher *Firmicutes*, and higher *Bacteroidetes* abundances than that of the preterm infant donor (Figure S1, Table S1).^30,31^ At an FDR threshold of 0.05, MTerm mice showed significantly lower *Actinobacteria* abundance by two weeks postnatal and significantly lower *Proteobacteria*, lower *Actinobacteria*, higher *Firmicutes*, and higher *Bacteroidetes* abundances than MPreterm mice by four weeks postnatal (Figure S2). PERMANOVA with treatment as the predictor showed a significant (*p* < 0.0001) separation between MTerm and MPreterm microbiome composition, while PERMANOVAs with age as the predictor revealed significant separations between the microbiomes of two-week-old and four-week-old mice in both the MTerm (*p* < 0.0001) and MPreterm (*p* = 0.00896) groups. Dimensionality reduction also demonstrated that the microbiomes of offspring mice clustered together with their respective dams (Figure S3).

### Colonization with the full-term infant microbiome improves cognitive function and reduces anxiety-like behaviors

We examined offspring behavior at four weeks of age. We did not find significant sex differences in any of the behavioral outcomes we measured (Table S2). We first assessed associative learning and memory in the MTerm and MPreterm groups using the contextual and cued fear conditioning test (*n*_*MTerm*_ = 13, *n*_*MPreterm*_ = 22). There was a significant difference in freezing time between MTerm (91.1 ± 28.99 s) and MPreterm (24.71 ± 4.56 s) mice in contextual testing (same environment and no acoustic conditioned stimulus (CS) presentation) (*p* = 0.0446). Furthermore, MTerm mice had significantly longer freezing time (147.8 ± 6.299 s) than MPreterm mice (126.1 ± 6.906 s) in the second half of the cued fear conditioning test (changed environment and acoustic CS presentation) (*p* = 0.0421) (Figure 1(b)).

We then employed the Morris water maze to assess spatial learning and memory in the two experimental groups (*n*_*MTerm*_ = 9, *n*_*MPreterm*_ = 16). MTerm and MPreterm mice both showed decreased latencies in finding the escape platform from days one to four (*p* < 0.0001), demonstrating learning in both groups. Notably, MTerm mice overall took less time to find the escape platform (*p* = 0.0465). There was no significant interaction (*p* = 0.539). These findings were determined by two-way ANOVA with predictors of training day and treatment (Figure 1(c)).

In an open field test to assess anxiety-like behaviors, MPreterm offspring demonstrated a reluctance to explore the open space (*n*_*MTerm*_ = 12, *n*_*MPreterm*_ = 22). They had significantly higher overall immobile time (63.85 ± 7.147 s) than that of MTerm (39.37 ± 6.859 s) mice at four weeks of age (*p* = 0.0276). Further analysis demonstrated that even though total time spent in the border zone of the open field was not significant between MTerm (1033 ± 16.74 s) and MPreterm (1042 ± 15.40 s) (*p* = 0.643), the immobile time in the border zone was significantly lower in MTerm (37.09 ± 6.89 s) than that of MPreterm (59.25 ± 6.495 s) mice (*p* = 0.0361). There was no significant difference in total distance traveled between the two groups (*p* = 0.120) (Figure 1(d)).

### Mice colonized with a full-term infant microbiome have decreased early-life BBB permeability

**Figure 2.**
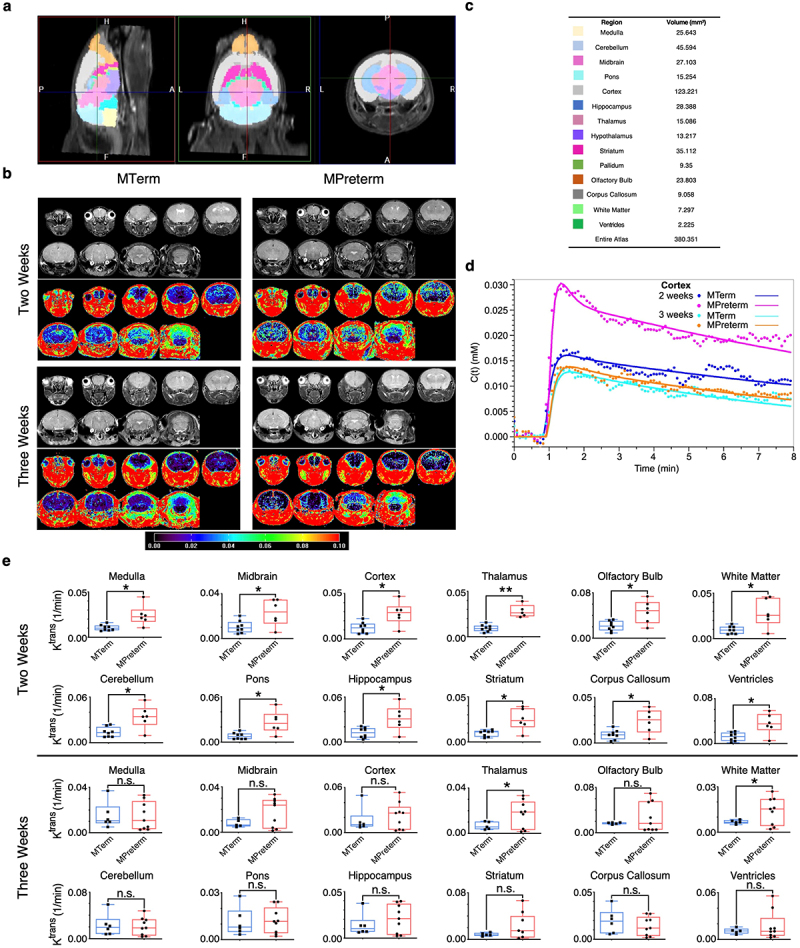
Mice colonized with the full-term infant microbiome display lower brain-wide BBB permeability in early life. (a) brain regional volume analysis was conducted using a 3D mouse brain atlas via VivoQuant. The 3D brain atlas is based on the Paxinos–Franklin atlas registered to a series of high-resolution MR images with 100 μm near-isotropic data using T2-w MRI. (b) Representative T2-w and T1-w images of MTerm (left) and MPreterm (right) mouse brains at two (top) and three (bottom) weeks of mouse age. Typical multi-slice T2-w mouse brain anatomic images (grayscale) alongside corresponding *K*^*trans*^ (min^−1^) T1-w colormaps. (c) Measurement of regional volume of the brain using VivoQuant mouse whole brain atlas. (d) Representative plots comparing contrast agent concentration curves *C(t)* as a function of time *t* obtained from the cortex for MTerm and MPreterm mice at two and three weeks of age. The gadolinium-based contrast agent omniscan (gadodiamide) was given by an intravenous bolus injection of ~0.2 mmol/kg body weight. (e) Brain-region-specific comparisons of BBB permeability at two and three weeks of age. The measurement of *K*^*trans*^ reflects the combined influence of various processes (primarily blood blow and capillary leakage) in these brain regions. n.s. not significant, * *p* < 0.05, ** *p* < 0.01, Welch’s *t*-test.

We then tested whether full-term and preterm microbiomes have different effects on BBB function via magnetic resonance imaging (MRI) of the MTerm and MPreterm mouse brain at both two and three weeks of age. To pinpoint brain regions susceptible to developmental BBB deficits, we created an automated segmentation protocol. Specifically, we non-linearly aligned the VivoQuant 3D mouse whole-brain atlas to the individual T2-weighted (T2-w) MR images, enabling precise definition and measurement of brain region volumes (Figure 2(a–b)). Using this method, we determined the volumes of 14 distinct brain regions (Figure 2(c)). These coordinates for the whole brain and sub-regions were subsequently applied to T1-weighted (T1-w) dynamic contrast-enhanced (DCE) MRI data (Figure 2(b)) to determine: i) the total and subregional brain volumes and ii) regions particularly vulnerable to BBB dysfunction in the developing brain. The volume of the pallidum was significantly (*p* = 0.0421) lower in MTerm than MPreterm mice at two weeks of age (*n*_*MTerm*_ = 8, *n*_*MPreterm*_ = 8). We detected no significant differences in brain regional volume at three weeks of age (*n*_*MTerm*_ = 7, *n*_*MPreterm*_ = 9) (Table S3).

However, our DCE-MRI analysis revealed that BBB permeability in twelve of the fourteen brain regions we examined during early development was significantly lower in MTerm mice. The DCE-MRI statistic *K^trans^* is the volume transfer constant (min^−1^) for the gadolinium-based contrast agent between blood plasma and the tissue extravascular extracellular space (EES), reflecting the combined processes (primarily blood flow and capillary leakage) that determine gadolinium influx rate from plasma into the EES. Higher values of *K^trans^* indicate greater vascular permeability. Based on the curves used to calculate *K^trans^* values (Figure 2(d)), we detected significant functional deficits in BBB permeability in MPreterm mice compared to MTerm mice at two weeks of age (*n*_*MTerm*_ = 8, *n*_*MPreterm*_ = 6). These deficits were observed in the medulla, midbrain, cortex, thalamus, olfactory bulb, white matter, cerebellum, pons, hippocampus, striatum, corpus callosum, and ventricles, though not the hypothalamus or pallidum. The thalamus and white matter, but no other brain regions analyzed, continued to show significantly lower *K*^*trans*^ values in three-week-old MPreterm mice (*n*_*MTerm*_ = 6, *n*_*MPreterm*_ = 9), demonstrating that microbiome-induced developmental BBB disruption occurs during early life (Figure 2(e)).

### Whole-brain scRNA-seq reveals extensive transcriptomic differences in BBB-relevant cell types in MTerm vs. MPreterm mice

**Figure 3.**
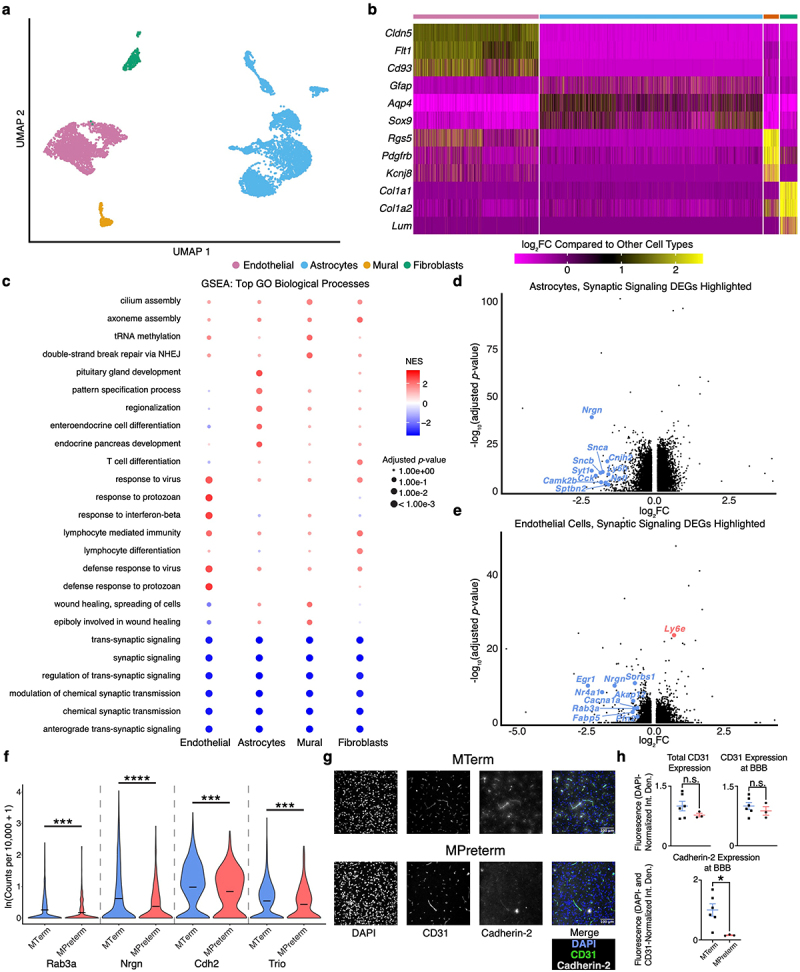
Mice colonized with the preterm infant gut microbiome show broad, coordinated disruption of BBB-relevant cell types at the transcriptomic level. (a) UMAP visualization of BBB-relevant cell types (subset and re-embedded using PCA, 50 dimensions). (b) Heatmap of canonical marker genes to confirm correct identification of each cell type. (c) Gene set enrichment analysis for BBB-relevant cell types. The top five upregulated GO biological processes by normalized enrichment score (NES) in MTerm and top five upregulated in MPreterm are plotted across all cell types. GSEA was performed using clusterProfiler with Benjamini–Hochberg *p*-value adjustment. “NHEJ” = nonhomologous end joining. Blue indicates enrichment in MTerm, red in MPreterm. (d–e) volcano plots of all genes with detectable expression in (d) astrocytes or (e) endothelial cells. The top ten DEGs by absolute value of log_2_FC with the GO annotation “synaptic signaling” are highlighted. (f) Violin plots show downregulation of “synaptic signaling”-annotated genes expressed in endothelial cells with established functions in BBB integrity. *** *p*_*adjusted*_ < 10^−3^, **** *p*_*adjusted*_ < 10^−4^, Seurat FindMarkers implementation of MAST. (g) Representative images of fluorescence microscopy of CD31 (green) (showing location of endothelial cells constituting BBB), Cadherin-2 (gray), and DAPI (nuclei, blue). ImageJ was used for analysis and quantification, and five to seven images were inspected for each mouse. Polygonal areas containing CD31 were created in ImageJ to identify BBB location (see Figure S5). (h) Cadherin-2 expression was significantly lower at the BBB of MPreterm mice. Top left: CD31 expression normalized to overall DAPI expression. Top right: CD31 expression normalized to DAPI expression within CD31+ areas. Bottom: cadherin-2 expression normalized to DAPI expression and CD31 expression within CD31+ areas. n.s. not significant, * *p* < 0.05, Wilcoxon rank sum test. Data presented as mean ± SEM.

Given the notable BBB functional defects we observed, we hypothesized that the altered gut microbiomes would result in distinct transcriptional profiles of BBB-relevant cell types. We, therefore, performed whole-brain scRNA-seq on MTerm and MPreterm mice at two weeks of age (*n*_*MTerm*_ = 4, *n*_*MPreterm*_ = 4). After preprocessing (see Methods), we clustered cells originating from both groups and annotated all major brain cell types (Figure S4) before focusing on four broad cell types known to be relevant to normal and perturbed BBB function: ECs, astrocytes, mural cells (comprising both pericytes and vascular smooth muscle cells), and fibroblasts (Figure 3(a)).^22^ We identified these cell types via their expression of canonical marker genes (Figure 3(b)). Using a minimum difference in expression of 30% (|log_2_FC| ≥ 0.3785) and a maximum adjusted *p-*value of 0.05, we identified 398 differentially expressed genes (DEGs) between MTerm and MPreterm ECs, 1,369 in astrocytes, 143 in fibroblasts, and 3 in mural cells (Table S4).

### MPreterm mice show broad downregulation of synaptic signaling pathways

To uncover broad processes that may participate in the effects of the microbiome on neurodevelopment, we performed gene set enrichment analysis (GSEA) on our scRNA-seq differential gene expression data. GSEA uncovered a complex reprogramming of the BBB following microbiome perturbation, with each cell type showing both unique and coordinated responses. In ECs, we observed significant upregulation of immune defense and interferon-beta signaling pathways in the MPreterm group. Mural cells from MPreterm mice showed significant enrichment in cilium assembly, tRNA methylation, and wound healing processes. Astrocytes from the MPreterm group demonstrated enrichment in cellular structural pathways and metabolic functions compared to those from MTerm mice. Fibroblasts displayed upregulation of axoneme assembly and immune functions such as lymphocyte-mediated immunity in MPreterm mice. Notably, across all cell types examined, synaptic signaling-related pathways showed strong negative enrichment scores, indicating consistent and robust downregulation in the MPreterm group. These synaptic pathways, including trans-synaptic signaling, chemical synaptic transmission, and modulation of chemical synaptic transmission, displayed some of the most significant enrichment patterns in the dataset (Figure 3(c), Table S5).

As downregulation of synaptic signaling represents a plausible mechanism of cognitive impairment, we decided to further explore the genes involved in this GSEA finding. We focused specifically on differential expression in astrocytes and ECs, as these cell types showed the largest transcriptomic differences between MTerm and MPreterm mice. We examined DEGs with a Gene Ontology (GO) annotation of “synaptic signaling” (GO:0099536) using the Jackson

Laboratory Mouse Genome Informatics database and found 82 such DEGs in astrocytes and 24 in ECs in our transcriptomic data. Here, we quantified the level of differential expression by log_2_FC, with negative values indicating downregulation and positive values indicating upregulation in MPreterm animals. In astrocytes, we identified striking downregulation in MPreterm compared to MTerm mice of *Nrgn* (log_2_FC = −2.11, *p*_*adjusted*_ = 1.06 × 10^−39^), *Camk2b* (log_2_FC = −1.80, *p*_*adjusted*_ = 2.13 × 10^−5^), and *Snca* (log_2_FC2 = −1.74, *p*_*adjusted*_ = 8.13 × 10^−11^), all of which are genes vital in the control of various synaptic functions.^32–34^ Other key genes downregulated in MPreterm astrocytes with “synaptic signaling” annotations include *Rab3a* (log_2_FC = −0.81, *p*_*adjusted*_ = 2.42 × 10^−15^) and *Syt1* (log_2_FC = −2.11, *p*_*adjusted*_ = 1.69 × 10^−11^). Both genes are involved in Ca^2+^-mediated vesicle exocytosis, meaning that astrocyte-neuron communication may be affected (Figure 3(d), Table S6).^35–38^

We further observed downregulation of several “synaptic signaling”-annotated genes in MPreterm ECs that also play roles in BBB integrity. *Rab3a* (log_2_FC = −0.70, *p*_*adjusted*_ = 1.50 × 10^−4^) and *Nrgn* (log_2_FC = −1.41, *p*_*adjusted*_ = 8.98 × 10^−11^) were both downregulated in MPreterm ECs. *Rab3a* is a regulator of endothelial lumen and tube formation, while *Nrgn* plays a critical role in controlling BBB permeability through Akt signaling.^39–41^ *Cdh2* (log_2_FC = −0.42, *p*_*adjusted*_ = 3.99 × 10^−4^) and *Trio* (log_2_FC = −0.38, *p*_*adjusted*_ = 6.56 × 10^−4^) were also “synaptic signaling”-annotated genes downregulated in MPreterm ECs. The Cadherin-2 and Trio proteins, likewise, contribute to BBB integrity by forming a complex that promotes the assembly and stabilization of VE-cadherin junctions between ECs.^42^ Both genes are also well-established regulators of synaptic transmission (Figure 3(e–f), Table S6).^43,44^ To validate these scRNA-seq findings, we performed an immunofluorescence assay using MTerm and MPreterm brain tissue, co-staining for CD31 (an endothelial cell marker) and Cadherin-2 (*n*_*MTerm*_ = 6, *n*_*MPreterm*_ = 3). As expected, we found no significant difference in CD31 expression but significantly (*p* < 0.05) higher Cadherin-2 expression at the BBB of MTerm mice compared to that of MPreterm mice (Figure 3(g–h), Figure S5).

### Microbiome maturity affects solute carrier expression in brain endothelial cells

Another crucial aspect of BBB function comes from its expression of transporters. Underexpression of transporters can impair the delivery of essential nutrients and removal of waste products, while overexpression can compromise the selectivity of the BBB. Many of these crucial transporters belong to the solute carrier (SLC) superfamily, which encompasses a diverse group of membrane proteins responsible for facilitating the movement of amino acids, neurotransmitters, metabolites, and many other solutes across cellular membranes.^45^ We observed transcriptional dysregulation of SLCs in the MPreterm endothelia. *Slc25a25* (log_2_FC = −1.17, *p*_*adjusted*_ = 0.00901) and *Slc38a2* (log_2_FC = −0.493, *p*_*adjusted*_ = 6.36 × 10^−8^) were both downregulated in MPreterm ECs. *Slc25a25* is a Ca^2+^-sensitive mitochondrial carrier that maintains ATP homeostasis by mediating the exchange of ATP- Mg^2+^ for inorganic phosphate across the inner mitochondrial membrane, and *Slc38a2* facilitates the net uptake of small neutral amino acids into the cell in an Na^+^-dependent manner.^46,47^ Conversely, *Slc29a4* (log_2_FC =0.651, *p*_*adjusted*_ = 0.00915) and *Slc16a2* (log_2_FC =0.459, *p*_*adjusted*_ = 1.23 × 10^−7^) were both upregulated in MTerm ECs. *Slc29a4* is a transporter of dopamine, serotonin, and other monoamine neurotransmitters, while *Slc16a2* mediates the movement of thyroid hormones (triiodothyronine and thyroxine) across cell membranes and is known to function at the BBB (Table S4).^48–51^

### Microbiome maturity drives distinct transcriptional states in brain endothelial cells and astrocytes

**Figure 4.**
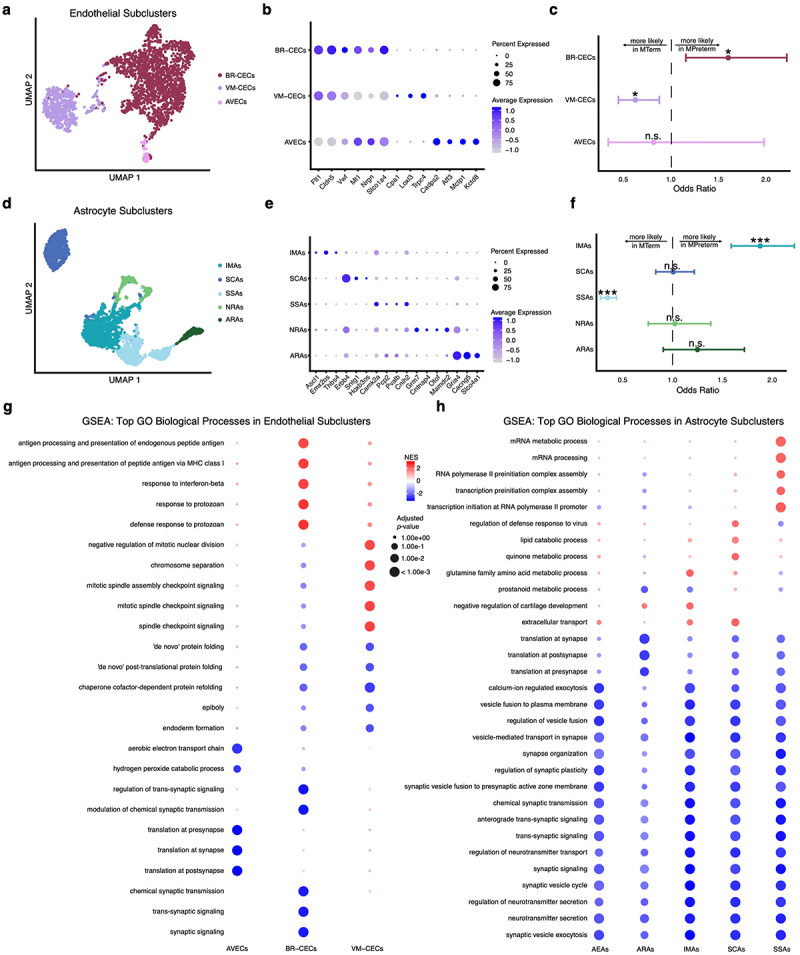
Microbiome maturity promotes distinct transcriptional states in brain endothelial cells and astrocytes. (a) UMAP plot for the three EC subclusters identified, named by the terms their marker genes in panel b suggest as appropriate: barrier-reactive capillary endothelial cells (BR-CECs), vaso-modulatory capillary endothelial cells (VM-CECs), and arterial and venous endothelial cells (AVECs). (b) Dot plot with relevant marker genes for each subcluster. The known functions of these marker genes were used to name the subclusters. Dot size represents the percentage of cells within a subcluster expressing a given gene, and dot color represents the average expression of that gene across the subcluster relative to other subclusters. (c) Odds ratios from logistic regression showing the relative likelihood of each EC subtype in MPreterm versus MTerm, controlling for sex. n.s. not significant, * *p*_*adjusted*_ < 0.05, ** *p*_*adjusted*_ < 0.01, *** *p*_*adjusted*_ < 0.001, bonferroni-corrected Wald test. Error bars represent 95% CI. (d) UMAP for the five astrocyte subclusters identified, named by the terms their marker genes in panel e suggest as appropriate: insult-mediating astrocytes (IMAs), synaptic connectivity astrocytes (SCAs), synaptic signaling astrocytes (SSAs), adhesion-enriched astrocytes (AEAs), and AMPA receptor-expressing astrocytes (ARAs). (E) same as panel b but for astrocyte subclusters. (f) same as panel g but for astrocyte subclusters. (g–h) gene set enrichment analysis for (g) EC and (h) astrocyte subclusters. The top five upregulated GO biological processes by NES in MTerm and top five upregulated in MPreterm are plotted across all subclusters. Blue indicates enrichment in MTerm, red in MPreterm,

We next investigated how microbiome maturity affects the distributions of transcriptional states in BBB cell populations by subclustering both MTerm and MPreterm ECs and astrocytes. We identified three endothelial subclusters and named them according to their gene markers: barrier reactive capillary endothelial cells (BR-CECs), vaso-modulatory capillary endothelial cells (VM-CECs), and arterial and venous endothelial cells (AVECs) (Figure 4(a)). The key markers indicating whether cells in each subcluster are of capillary or arterial/venous origin were established by Yang and colleagues^52^ and validated by Bryant et al.^20^ The BR-CEC and VM-CEC markers distinguish cells in distinct transcriptional states primarily through the expression of transport-related genes (like *Slco1a4*, a solute carrier transporter, and tight junction protein *Cldn5*) in BR-CECs versus vasculature-regulating genes (like *Loxl3*, involved in crosslinking the extracellular matrix, and *Trpc4*, a calcium channel) in VM-CECs (Figure 4(b)).^53–56^ Using a logistic regression model, we found that AVECs are not significantly enriched in either condition but that cells in the BR-CEC transcriptional state are significantly more likely to originate from MPreterm mice (OR = 1.60, *p*_*adjusted*_ = 0.015), and cells in the VM-CEC cluster are significantly enriched in MTerm mice (OR = 0.62, *p*_*adjusted*_ = 0.019) (Figure 4(c)).

The microbiome similarly drove substantial transcriptional reprogramming in astrocyte populations across the brain. Subclustering analysis revealed five functionally distinct astrocyte transcriptional states: insult-mediating astrocytes (IMAs), synaptic connectivity astrocytes (SCAs), synaptic signaling astrocytes (SSAs), adhesion-enriched astrocytes (AEAs), and AMPA

receptor (AMPAR)-expressing astrocytes (ARAs) (Figure 4(d)). The five astrocyte populations revealed distinct functional identities through their gene expression profiles. IMAs were characterized by the expression of developmental regulator *Ascl1* and extracellular matrix protein *Thbs4*, which are both well-known to be upregulated in response to CNS injury.^57,58^ SCAs expressed *Erbb4*, a receptor tyrosine kinase, and scaffolding protein *Sntg1*.^59,60^ SSAs were distinguished by markers *Camk2a*, which encodes the α subunit of calmodulin-dependent kinase II, and *Cnih2*, which regulates AMPAR assembly, highlighting their role in synaptic function.^61–63^ Finally, AEAs displayed adhesion molecules *Mamdc2* and *Cntnap4*, while ARAs had marker genes *Gria4*, which encodes the AMPAR subunit GluR4, and *Cacng5*, which encodes an auxiliary AMPAR subunit and modifies its activity (Figure 4(e)).^64–68^ Logistic regression analysis revealed that IMAs are significantly enriched in MPreterm mice compared to controls (OR = 1.88, *p*_*adjusted*_ = 7.98 × 10^−13^), while SSAs are significantly more abundant in MTerm mice (OR = 0.35, *p*_*adjusted*_ < 10^−16^) (Figure 4(f)).

Gene set enrichment analysis of endothelial and astrocyte subclusters revealed distinctive functional adaptations in response to microbiome immaturity, providing further resolution to analyze perturbed pathways. In MPreterm mice, BR-CECs exhibited enrichment of immune-related pathways, including interferon-beta signaling and MHC class I-mediated antigen presentation. MPreterm VM-CECs, on the other hand, were enriched for processes related to mitosis and chromosomal dynamics like spindle checkpoint signaling and chromosome separation but downregulation of processes related to *de novo* protein folding and development. Both AVECs and BR-CECs exhibited downregulation of processes related to synaptic transmission in MPreterm mice, while VM-CECs did not. All MPreterm astrocyte subclusters, though, exhibited notable downregulation of a diversity of synaptic signaling- related processes including regulation of vesicle fusion, regulation of neurotransmitter transport, and synaptic vesicle exocytosis. MPreterm SSAs were also enriched for terms related to transcription and mRNA processing (Figure 4(g–h), Table S7).

### Full-term- and preterm-associated bacterial communities exhibit distinct metagenomic taxonomies, functional genomic pathways, and metabolomic profiles

**Figure 5.**
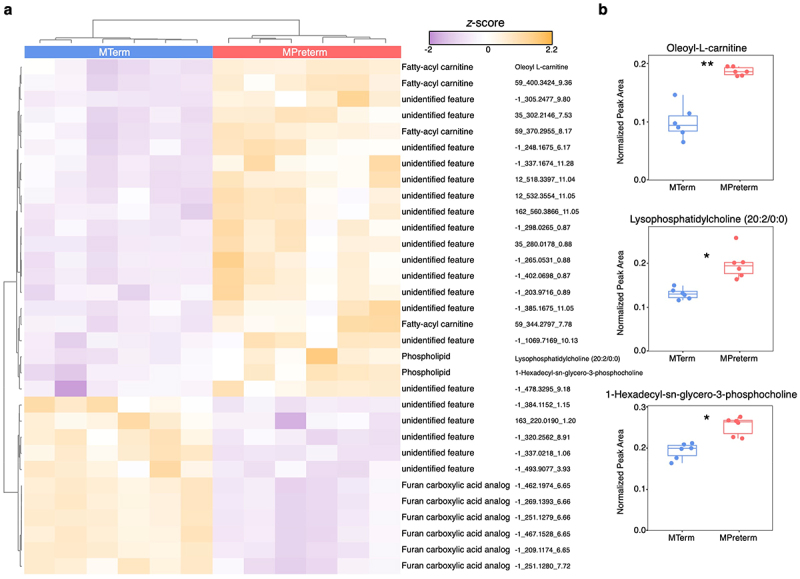
Microbial metabolite acylcarnitines and phospholipid derivatives are present at diminished levels in MTerm serum. (a) Heatmap of the 32 serum microbial metabolites present at significantly (FDR threshold = 0.05) different levels in MTerm and MPreterm. Purple represents low abundance of a metabolite, and yellow represents high abundance. The plot was generated using Euclidean distance with Ward clustering. Panel adapted from MetaboAnalyst. (b) Comparison of the three putatively identified metabolites present at significantly different levels. * *p*_*adjusted*_ < 0.05, ** *p*_*adjusted*_ < 0.01, Benjamini–Krieger–Yekutieli-adjusted unpaired *t*-test.

Whether or which microbial metabolites reach the CNS to have an impact on brain function is dependent on the systemic communication between peripheral blood and the tightly regulated BBB. In addition, these metabolites might directly affect BBB development and function, and thereby indirectly regulate CNS functions, without even entering the CNS. To dissect the metabolic features of the MTerm and MPreterm microbial communities that may affect cognition and BBB function, we analyzed serum samples from both groups at four weeks of age via untargeted metabolite profiling (*n*_*MTerm*_ = 6, *n*_*MPreterm*_ = 6). The metabolomics experiment resulted in 1,571 features captured, of which 271 could be putatively identified and were not known mass spectrometry (MS) contaminants (Table S8). There was a distinct compositional difference in the serum samples between treatments, as shown in the heatmap of 32 features with significantly differential abundance (FDR threshold = 0.05) (Figure 5(a)). Of these features, three could be putatively identified. All were significantly lower in the MTerm serum: oleoyl-L-carnitine (from NIST14_426.3581_9.56), lysophosphatidylcholine (20:2/0:0) ([M+H]+ C28H55N1O7P1_548.3714_9), and 1-hexadecyl-sn-glycero-3-phosphocholine (from NIST14_482.3609_9.42) (Figure 5(b)).

**Figure 6.**
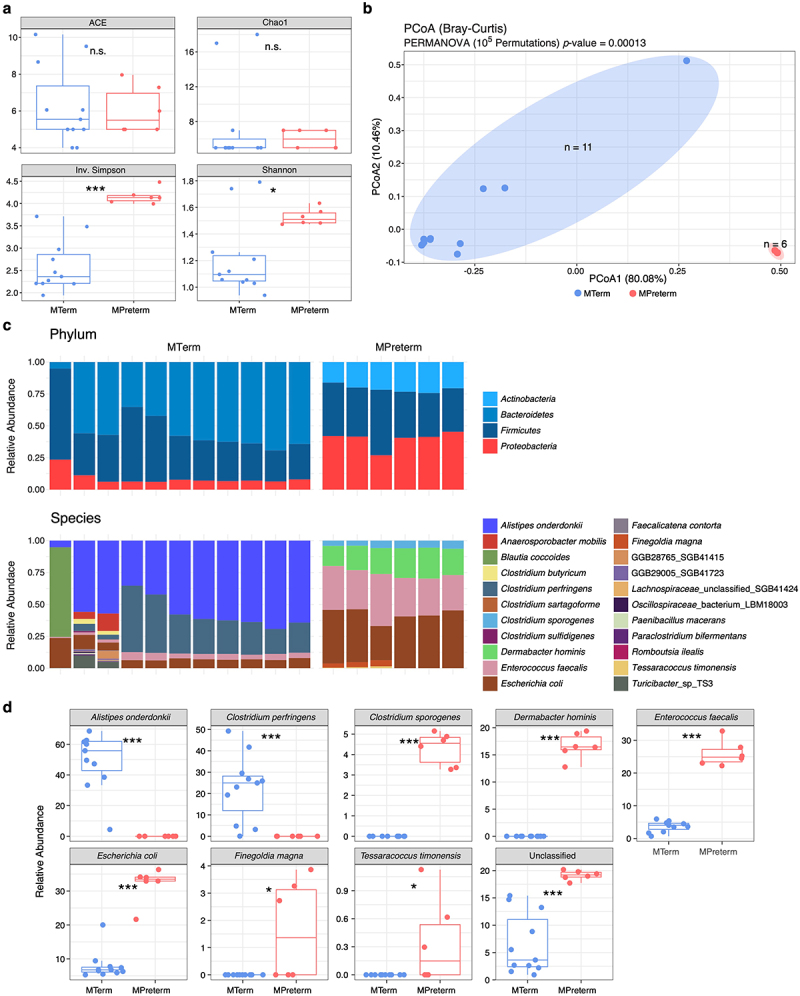
MTerm and MPreterm gut microbiomes differ with respect to bacterial diversity and composition. n.s. not significant, * *p*_*adjusted*_ < 0.05, ** *p*_*adjusted*_ < 0.01, *** *p*_*adjusted*_ < 0.001, Benjamini–Hochberg-adjusted Wilcoxon rank sum test. (a) Comparison of α-diversity metrics. ACE and Chao1 measure richness, while inverse Simpson and Shannon measure both evenness and richness. (b) Principal coordinate analysis (PCoA) of MTerm and MPreterm microbiomes. (c) Comparisons at the phylum and species level of bacterial compositions of the gut microbiome. One column represents one mouse. (d) Bacterial species present at altered levels between MTerm and MPreterm mice are shown.

To identify which metagenomic pathways and associated species might contribute to the distinct metabolite profiles observed between the MTerm and MPreterm mice, we subjected fecal samples from both groups at four weeks of age to shotgun metagenomic sequencing (*n*_*MTerm*_ = 11, *n*_*MPreterm*_ = 6). We first measured the α-diversity (within-sample diversity) of the microbial communities. Richness metrics (Chao1 and ACE) showed no significant differences between groups. However, Inverse Simpson (*p* < 0.001) and Shannon (*p* < 0.05), diversity metrics accounting for both richness and evenness, were significantly lower in MTerm offspring (Figure 6(a,c)). For β-diversity, which quantifies between-sample differences in microbial community composition, PERMANOVA with treatment as the predictor revealed significant separation between groups (*p* = 0.00013), using Bray-Curtis dissimilarity based on all operational taxonomic units (Figure 6(b–c)). At the phylum level, the relative abundances of *Actinobacteria* and *Proteobacteria* were significantly decreased while *Bacteroidetes* was significantly enriched in the MTerm group compared to the MPreterm group (FDR threshold = 0.05) (Figure 6(c)). At the species level, MTerm mice had significantly lower abundances of *Clostridium sporogenes*, *Dermabacter hominis*, *Enterococcus faecalis*, *Escherichia coli*, *Finegoldia magna*, and *Tessaracoccus timonensis*, and significantly greater abundances of *Alistipes onderdonkii* and *Clostridium perfringens*, when compared to MPreterm mice (FDR threshold = 0.05) (Figure 6(d)).

**Figure 7.**
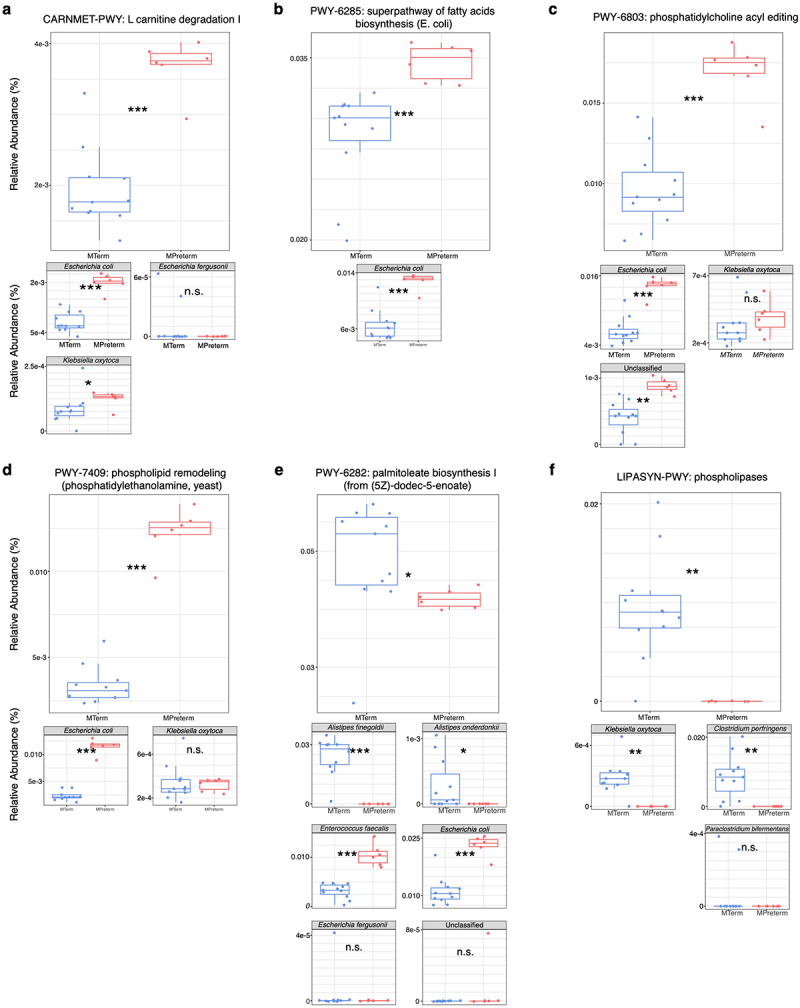
Metagenomic pathways present at different levels in MTerm and MPreterm microbiomes are associated with disparate abundances of key bacterial strains. the pathways present here are those corresponding to production and processing of oleoyl-L-carnitine, lysophosphatidylcholine (20:2/0:0), and 1-hexadecyl-sn-glycero-3-phosphocholine, the metabolites we identified at lower levels in MTerm mice. Below each pathway boxplot are boxplots showing the respective abundances of bacterial strains possessing the relevant genomic pathway. n.s. not significant, * *p*_*adjusted*_ < 0.05, ** *p*_*adjusted*_ < 0.01, *** *p*_*adjusted*_ < 0.001, Benjamini–Hochberg-adjusted Wilcoxon rank sum test. (a) “CARNMET-PWY: L-carnitine degradation I”. (b) “PWY-6284: superpathway of fatty acids biosynthesis (E. coli)”. (c) “PWY-6803: phosphatidylcholine acyl editing”. (d) “PWY-7409: phospholipid remodeling (phosphatidylethanolamine, yeast)”. (e) “PWY-6282: palmitoleate biosynthesis I (from (5Z)-dodec-5-enoate)”. (f) “LIPASYN-PWY: phospholipases.”

A total of 291 metagenomic pathways showed significant differences between MTerm and MPreterm groups (FDR threshold = 0.05) (Table S9). These pathways primarily involved amino acid, carbohydrate, lipid, and nucleotide metabolism, cell structure components, and biosynthesis of cofactors and vitamins. Many of the remaining pathways were related to energy metabolism. Corresponding to the higher levels of oleoyl-L-carnitine in MPreterm serum, “CARNMET-PWY: L-carnitine degradation I” was significantly enriched in the MPreterm group. The species enriched in the MPreterm microbial community associated with this pathway were *Escherichia coli* and *Klebsiella oxytoca* (Figure 7(a)). Several pathways were enhanced in relation to increased levels of lysophosphatidylcholine (20:2/0:0) and 1-hexadecyl-sn-glycero-3-phosphocholine, including “PWY-6284: superpathway of fatty acids biosynthesis (E. coli)” (Figure 7(b)), “PWY-6803: phosphatidylcholine acyl editing” (Figure 7(c)), and “PWY-7409: phospholipid remodeling (phosphatidylethanolamine, yeast)” (Figure 7(d)). These pathways were associated with increased abundance of *Escherichia coli* in the MPreterm group. Higher levels of lysophosphatidylcholine (20:2/0:0) and 1-hexadecyl-sn-glycero-3-phosphocholine in the MPreterm group also corresponded to a decrease in the pathway “PWY-6282: palmitoleate biosynthesis I (from (5Z)-dodec-5-enoate).” This corresponded with reduced abundance of *Alistipes finegoldii* and *Alistipes onderdonkii* and increased abundance of *Enterococcus faecalis* and *Escherichia coli* in the MPreterm microbiome (Figure 7(e)). Finally, higher serum levels of these two metabolites in MPreterm mice were associated with a decreased presence of the “LIPASYN-PWY: phospholipases” metagenomic pathway and decreased abundance of *Klebsiella oxytoca* and *Clostridium perfringens* (Figure 7(f)).

## Discussion

The gut microbiome is increasingly recognized as an important factor in the etiology of neurological disease.^5–10^ Previous observational research has identified a correlation between an altered microbiome and worsened neurodevelopmental outcomes, and some experimental work has hinted at a relationship between gut microbiota and brain development in a maternal-fetal model and a preterm infant context.^69–73^ In particular, we have previously shown that the maturation of the preterm-associated microbiome during early development establishes a foundation that influences associative memory and learning capabilities in adulthood.^17^ Nonetheless, the precise mechanisms underlying the remarkable effects of the gut microbiome on neurodevelopment are yet to be delineated. Here, we have shown in a humanized, gnotobiotic mouse model that transplantation with the full-term, as opposed to preterm, infant gut microbiome fosters normal cognitive function, maintains early-life BBB integrity, and promotes BBB-derived control of synaptic signaling, potentially through the mediation of microbiome-derived systemic metabolites.

The behavioral outcomes we observed in mice harboring the full-term microbiome highlight its critical role in supporting healthy cognitive and emotional development. Specifically, preserved associative fear learning and reduced anxiety-like behaviors in MTerm mice indicate healthy hippocampal and amygdala function, consistent with previous studies linking altered microbiome composition to impaired working memory, disrupted stress responsivity, decreased sociability, and reduced cognitive flexibility.^71–78^ The enhanced spatial and fear learning performance by MTerm mice suggests improved development of neural circuits involved in memory consolidation and retrieval, possibly supported by synaptic plasticity due to functional astrocyte-neuron interactions. This is reinforced by our transcriptomic data and prior evidence linking microbiome perturbations to changes in synaptic connectivity.^73,78^ Collectively, these behavioral phenotypes imply that microbiome maturity is essential for establishing and maintaining proper developmental trajectories of neural circuits underlying cognitive processing and emotional regulation.

Our key observation of lower early-life BBB permeability in MTerm mice suggests that the BBB is likely a critical interface regulating the gut-brain axis in development. Our findings are consistent with previous work by Braniste and colleagues, who demonstrated that germ-free mice display greater BBB permeability than specific pathogen-free controls, and we extend this to a direct comparison of the effects of mature and immature human-derived microbiomes.^25^ In MTerm mice, we found decreased permeability across nearly all brain regions in our experiment (measured by *K*^*trans*^ values) at two weeks of age, which persists in the thalamus and white matter, but not the rest of the brain, at the three-week mark. This points to the existence of a critical developmental window during which gut microbiota can influence BBB function and mirrors previous work which found that inflammatory insults increase BBB permeability in early-life rodents.^79,80^

Accordingly, early postnatal life represents a critical period for the consolidation of the maintenance mechanisms of the BBB. Most of the core structural development of the brain endothelium occurs embryonically, with tight junction proteins present at embryonic day 13 in the mouse brain and week eight of gestation in the human brain, but astrocytes, pericytes, and the basement membrane are still maturing.^81^ Substantial astrocyte differentiation and polarization, along with ensheathment of the neurovasculature by the astrocyte end-feet, occur during the first three weeks of postnatal life in rodents.^82–85^ In humans, astrocyte end-feet only cover approximately 60% of the brain microvasculature at birth compared to 80–90% in adults.^86,87^ Furthermore, the basement membrane thickens substantially during early postnatal development, and pericytes transition from having cell bodies that thoroughly enwrap the endothelium at birth to having extensive processes but only partial endothelial coverage by adulthood.^88–90^ These developmental processes may explain why permeability differences between the MTerm and MPreterm BBB are profound when measured at two weeks postnatal but largely disappear by three weeks of age, in agreement with previous literature which measured whole-brain BBB permeability.^79,80^ We were, however, able to detect differences in regional BBB permeability in the thalamus and white using our advanced DCE-MRI measurements that persisted in three-week-old mice. Importantly, in humans, increased BBB permeability in the white matter is associated with cognitive impairment.^91–93^ As such, our findings underscore the need for further investigation of key aspects of postnatal BBB development like astrocyte and pericyte coverage.

While we cannot definitively establish that the microbiome-induced transient BBB differences directly cause the observed behavioral changes, as these could represent parallel developmental processes, recent evidence suggests a plausible mechanistic connection. Dion-Albert *et al*. demonstrated through virus-mediated knockdown that compromising the BBB in the prefrontal cortex of female mice is sufficient to induce anxiety-like, depression-like, and social avoidance behaviors.^94^ Likewise, Greene *et al*., using similar methods, found that BBB disruption in the medial prefrontal cortex impaired spatial memory and object recognition, while such disruption in the hippocampus decreased grooming behavior and social novelty preference, though enhanced forced swim test performance.^95^ Moreover, there is a strong correlation between early-life microbiome maturity and fear learning ability in adulthood among mice harboring gut microbiota from preterm infants of various postmenstrual ages.^17^

If microbiome-induced early-life BBB disruption indeed leads to lasting behavioral changes, then there are several important translational implications. First, it suggests that therapeutic strategies aiming to improve neurodevelopmental outcomes of preterm infants should be administered in very early life to have maximum possible efficacy. By targeting the early immature microbiome by maternal probiotics or direct supplementation, there may be improvements in the function of the developing BBB that lead to improved long-term neurodevelopmental outcomes.^27,96,97^ Our results also imply that DCE-MRI might serve as a diagnostic tool of BBB dysfunction in early life. It has previously been used to examine BBB permeability in neurodegenerative diseases like Alzheimer’s and Parkinson’s.^98,99^ Extending its use to preterm infants could allow for region-specific analysis of BBB function and, thus, better prediction and management of neurodevelopmental outcomes.

Our scRNA-seq findings provide clarity into how microbiome maturity may drive neurodevelopment via BBB function. Broad transcriptional differences occur in MTerm vs. MPreterm ECs, astrocytes, mural cells, and fibroblasts, with each cell type exhibiting a distinct signature of affected pathways. Still, all four cell types converge on downregulation in the MPreterm brain of pathways related to the control of synaptic signaling, a crucial process for all of brain development and cognition. This implies that the neurovascular interactions essential for normal brain maturation are impaired in the MPreterm brain.^100–103^ Intriguingly, BBB dysfunction-associated disruption to synaptic signaling is also present in Alzheimer’s Disease, raising the possibility that parallel pathways contribute to both neurodevelopmental and neurodegenerative conditions.^104^ Importantly, the GO term “synaptic signaling” captures genes involved in neurotransmitter-mediated communication that, while largely originally characterized in neurons, extend far beyond classical synapses. This is because many similar molecular mechanisms are employed by non-neuronal cells for intercellular communication and signal transduction. For example, *Itpr1*, downregulated in MPreterm astrocytes (Table S4), mediates Ca^2+^ release from the endoplasmic reticulum in response to extracellular stimuli like ATP in astrocytes, while serving similar fundamental functions in neuronal synaptic plasticity.^105–107^

Subcluster analysis of ECs and astrocytes provided further clarity, revealing that preterm microbiome-exposed mice had a significant enrichment of barrier-reactive capillary endothelial cells, characterized by heightened immune activation and stress responses. This likely represents a compensatory mechanism to repair compromised barrier function. Conversely, the significant depletion of vaso-modulatory capillary endothelial cells in these mice suggests a failure to sustain normal BBB homeostasis. MPreterm astrocytes similarly exhibited distinct transcriptional shifts, with a pronounced increase in insult-mediating astrocytes at the expense of synaptic signaling astrocytes, highlighting an inflammatory cellular milieu plausibly detrimental to synaptic health and plasticity. Finally, our finding of lower protein expression of Cadherin-2, but not CD31, at the MPreterm BBB compared to that of MTerm mice suggests that the immature microbiome primarily affects the formation of the intercellular junctions between ECs, not the total number of ECs. Thus, our transcriptomic analysis of the MPreterm brain strongly supports a model in which immature-microbiome-driven transcriptional alterations within BBB-associated cells disrupts their roles in supporting neurodevelopment.

The metagenomics analysis we conducted reveals a compelling potential mechanistic link between microbiome maturity and neurodevelopment through metabolite profiles. The enrichment of specific beneficial commensals like *Alistipes onderdonkii* and the suppression of potentially harmful bacterial species in MTerm mice such as *Escherichia coli*, *Enterococcus faecalis*, and *Clostridium sporogenes* reflects a balanced community that promotes healthy host metabolism. Moreover, our finding of higher *Proteobacteria* abundance in the microbiome of MPreterm compared to MTerm mice is consistent with previous work that found that *Proteobacteria* is a dominant phylum in the preterm infant microbiome.^30,108,109^ The lower α-diversity metrics in MTerm offspring indicates a more functionally coherent microbial community. Multiple lines of evidence support the importance of these microbial profiles in neurodevelopment. For example, *E. coli* infection in early life can both disrupt microbiome composition and lead to cognitive impairment.^110,111^ *Alistipes* bacteria, in contrast, are present at decreased levels in autism spectrum disorder patients, suggesting its enrichment in MTerm mice may contribute to the stronger cognitive function we observed.^112,113^ However, the *Alistipes* genus was only identified relatively recently, meaning direct evidence of its role in neurodevelopment is lacking.

The decreased oleoyl-L-carnitine levels we found in MTerm mice are plausibly related to healthy neurodevelopment. Lower concentrations of long-chain acylcarnitines in the blood indicate preserved efficiency in the mitochondrial β-oxidation pathway and broader energy metabolism. Heightened long-chain acylcarnitine levels are often found in Alzheimer’s patients, demonstrating that disrupted energy homeostasis can be associated with cognitive impairment.^114^ Some previous research suggests acylcarnitines are also upregulated in preterm infants, though findings across studies are inconsistent.^115–117^ Our detection of decreased oleoyl-L-carnitine in MTerm mice suggests that maintaining lower long-chain acylcarnitine levels could serve as a metabolic biomarker of the influence of the full-term infant microbiome. This metabolic stability likely supports BBB integrity through limiting oxidative stress and more broadly enhancing the energy-intensive process of brain development.^118,119^ Furthermore, the lower phospholipid metabolite levels we observed in MTerm mice may also help to prevent BBB developmental disruption. Lysophosphatidylcholine (20:2/0:0) and 1-hexadecyl-sn-glycero-3-phosphocholine are both part of the broader lysophosphatidylcholine (lyso-PC) class of molecules. Administration of lyso-PCs *in vitro* induces oxidative stress and inflammation in ECs, providing a conceivable mechanism by which reduced concentrations of these microbial metabolites in MTerm mice can preserve BBB function.^120,121^ In fact, both oxidative stress and inflammation can alter the expression of SLC transporters, potentially in accordance with our scRNA-seq findings.^122,123^

While the gnotobiotic mouse model we used in our study allows for controlled experimentation, we acknowledge its limitations. First, the human neurodevelopmental trajectory differs from that of the mouse substantially, particularly with respect to cortical complexity, potentially limiting direct clinical translation.^124–127^ The timing of the untargeted metabolomics experiment presents another important limitation. This assay was conducted at four weeks postnatal, while we observed differences in BBB permeability between MTerm and MPreterm mice at two and three weeks postnatal. As such, though lyso-PCs can induce oxidative stress and inflammatory injury in ECs, both of which can damage the BBB, we cannot determine whether the specific metabolites we identified affected BBB integrity.^119,128^ Finally, our data demonstrate that MTerm mice display improved cognitive function and that the immature microbiome fails to mature the BBB in ways supported by the full-term microbiome. Though it is well established that increased BBB permeability can induce behavioral deficits, we have not shown a direct causal relationship between BBB function and the neurodevelopmental differences we observed between MTerm and MPreterm mice.^94,95,129^ To address these limitations, future studies should attempt to further correlate the relative abundances of specific gut microbiota species and neurodevelopmental outcomes in human infants, profile the metabolome from early infancy, and perform knockdown and rescue experiments of specific genes and metabolites with known functions at the BBB that we found present at altered levels between MTerm and MPreterm mice.

Taken together, our findings demonstrate that an immature microbiome is insufficient to maintain neurodevelopmental homeostasis, with BBB dysfunction and alterations to BBB regulation of synaptic signaling due to an altered microbial metabolite profile emerging as probable mechanisms. Our results provide mechanistic insight into why preterm birth often leads to cognitive impairment, identifying the immature microbiome as a key contributor. Importantly, we have identified specific bacterial populations and metabolic pathways as potential therapeutic targets. Manipulating the preterm infant microbiome through targeted probiotics, metabolite supplementation, or microbiome-directed dietary interventions could help restore normal BBB development and potentially mitigate long-term neurodevelopmental deficits. As such, future work should explore the reversibility of the effects we observed in MPreterm mice and determine whether it is possible to reshape the immature microbiome toward a state more conducive to normal brain development.

## Methods

### Human samples

This study was conducted with the approval of the Institutional Review Board of the University of Chicago (Protocol ID: IRB210270), which follows the Declaration of Helsinki and the Belmont Report. The parents of the infants enrolled in the study gave informed consent. The fecal sample donors were a full-term, healthy infant at GA of 39 weeks born via vaginal delivery (MTerm) and a preterm infant at GA of 24 weeks born via cesarean section (MPreterm). Patient post-meconium fecal samples provided at three weeks postnatal were obtained from the Center for the Science of Early Trajectories biorepository at the University of Chicago Comer Children’s Hospital, which operates a level IV NICU in Chicago, IL, USA. The infants enrolled had no congenital anomalies.

### Animal use

This study was conducted with the approval of the Institutional Animal Care and Use Committee of the University of Chicago. All animal experiments complied with the *Guide for the Care and Use of Laboratory Animals* (National Research Council (USA), 2011). All mice in this study were allowed *ad libitum* access to NIH-31 germ-free chow and water. Germ-free C57BL/6J mice (Jackson Laboratory, Bar Harbor, ME, USA) were maintained at the Gnotobiotic Research Animal Facility at the University of Chicago, where animals are routinely tested for microbes and parasites to ensure they remain germ-free. A mixture of female and male mice was used for all analyses. Sex differences in mouse behavior are strain-dependent and were not statistically significant in this study (Table S2).^130–134^ Exact sample sizes and breakdowns by treatment, age, and sex for each experiment are available in Table S10. Information on the use of mice from different litters is available in Table S11.

### Microbiota transplantation

The microbiota transplantation protocol used for this study was followed as previously described.^135^ 100 mg aliquots of the fecal samples from each donor were thawed. Under anaerobic conditions, fecal samples were resuspended in PBS, homogenates were clarified through 100 µm nylon filters and stored in PBS containing glycerol (concentration of 15% v/v) at −80°C in 600 µL aliquots. Germ-free mice were colonized by gavaging eight- to nine-week-old pregnant females (estimated embryonic day 15–17) with 250 µL aliquots of the prepared MTerm or MPreterm fecal supernatant. Pups in both the MTerm and MPreterm groups were delivered spontaneously and full-term and kept with their mothers until weaning at three weeks of age, allowing them to naturally acquire the transplanted patient-derived microbiota.

### Behavioral testing setup

Behavioral tests were conducted with both MTerm and MPreterm mice at four weeks of age. Trials were recorded and analyzed with ANY-Maze software (Stoelting Co., Wood Dale, IL, USA).

### Fear conditioning

The same procedure previously described for the contextual and cued fear conditioning test was employed here.^72^ Mice underwent fear conditioning involving pairings of a CS of a 50 dB white noise for 30 s and an unconditioned stimulus (US) of a 0.3 mA foot shock during the final 2 s of CS. On the conditioning day, mice freely explored the chamber for 2 min before experiencing three CS-US pairings at 2 min, 4 min, and 6 min, then remained undisturbed for an additional 90 s. After 24 h, mice were tested for contextual fear by being returned to the original chamber for 5 min without stimuli. 2 h later, cued fear was tested by placing mice in a novel environment for 6 min, with the CS presented during the last 3 min. Fear memory was quantified by measuring freezing duration during the contextual test and the final 3 min of the cued test.

### Morris water maze

Mice were placed in a circular tank (120 cm in diameter) filled with room-temperature water (22 °C) that had been made opaque by adding nontoxic white tempera paint. High-contrast black and white images were positioned on curtains surrounding the tank to provide visual reference points. During the training phase, mice learned to escape onto a visible 10 cm diameter platform that extended 1 cm above the water surface. This phase consisted of five trials, with the platform location changed between each trial. In the testing phase, mice were challenged to locate a hidden platform submerged 1 cm below the water surface in the southeast quadrant. Testing occurred over four consecutive days, with sessions conducted 24 h apart. Performance was measured by recording the time it took mice to find the escape platform. If a mouse failed to reach the platform within 60 s, a maximum score of 60 s was recorded.

### Open field test

Mice were placed individually in the center of an open field box. Their following measures of spontaneous motor activity were recorded: time spent immobile in the center and border zones, total time in the center and border zones, and distance traveled.

### Magnetic resonance imaging setup

All imaging experiments were performed at the University of Chicago Integrated Small Animal Imaging Research Resource. MTerm and MPreterm mice of two and three weeks of age were used. Mice underwent anesthesia before the experiments and were maintained on 1.5–2.5% isoflurane. Temperature, as well as heart and respiration rates, were monitored using a fiber optic detection system (SA Instruments, Stony Brook, NY, USA). A 9.4 T small animal scanner equipped with 11.6 cm inner diameter actively shielded gradient coils (maximum constant gradient strength across all axes: 230 mT/m) was used. Each mouse was positioned supine on an animal holder and placed into a 30 mm diameter quadrature volume coil (Rapid MR International, Columbus, OH, USA).

### T2-weighted imaging

Whole-brain coverage was achieved using multi-slice spin echo T2-w imaging in the coronal direction with a Rapid Acquisition with Relaxation Enhancement (RARE) pulse sequence (repetition time (TR) = 4000 ms, echo time (TE) effective = 20 ms, field-of-view (FOV) = 25.6 × 19.2 mm^2^, matrix size = 256 × 192, slice thickness = 0.5 mm, RARE factor = 4, number of excitations (NEX) = 2). Brain regional volume analysis utilized a 3D mouse brain atlas from the VivoQuant (Perceptive, Nottingham, UK) software. This 3D brain atlas is based on the Paxinos–Franklin atlas registered to high-resolution MRI with 100-μm near-isotropic data.^136,137^ This yielded 14 brain regions of interest (ROIs): medulla, cerebellum, midbrain, pons, cortex, thalamus, hypothalamus, hippocampus, striatum, pallidum, olfactory bulbs, corpus callosum, white matter, and ventricles.

### Native T1 mapping

A RARE-VTR (variable TR) pulse sequence was used (TR = 281, 350, 500, 1000, 1500, 2000, 3000, 5000, 10000 ms, TE = 12.3 ms, RARE factor = 4, FOV = 25.6 × 19.2 mm^2^, matrix size = 128 × 96, thickness = 1.5 mm, number of slices = 9, NEX = 1).

### Dynamic contrast-enhanced magnetic resonance imaging

DCE-MRI was used to model the movement of gadolinium contrast in the brain as a method to measure BBB permeability. T1-w DCE-MRI data (TR/TE = 52.05/1.56 ms, FOV = 25.6 × 19.2 mm^2^, matrix size = 128 × 96, flip angle = 30°, slice thickness = 1.5 mm, number of slices = 9) were acquired with a temporal resolution of 5.0 s. The DCE-MRI data were continually acquired before (for 1 min), during, and after a bolus intravenous injection of ~0.2 mmol/kg (body weight) of Omniscan (generic name: gadodiamide) (GE HealthCare, Chicago, IL, USA) for a total duration of 8 min (96 frames).

DCE-MRI data were analyzed in MATLAB. Specifically, the 14 brain ROIs obtained from T2-w images were applied to the DCE-MRI to determine the permeability of BBB at these regions. The contrast agent concentration curve *C(t)* as a function of time *t* was calculated using a previously published method based on the gradient echo signal equation (non-linear model).^138^ The standard Tofts model was used to fit *C(t)* to obtain maps of physiological parameters of the volume transfer constant *K*^*trans*^ between blood plasma and the extravascular extracellular space. The arterial input function was traced from the internal carotid artery.

### Data collection for whole-brain single-cell RNA sequencing

Whole-brain samples were collected from MTerm and MPreterm mice at two weeks of age. Before tissue dissociation, the meninges were removed. To isolate brain cells, the Neural Tissue Dissociation Kit – Postnatal Neurons (Miltenyi Biotec, Bergisch Gladbach, Germany) was used. The manufacturer’s protocol was followed with minimal modifications. First, a 40 µm strainer was used instead of a 70 µm one. This allowed for more precise filtering out of debris and, thus, better cell isolation. Moreover, the enzymatic tissue digestion incubation times were decreased from 15 to 8 min during the initial dissociation and from 10 to 6 min during the secondary dissociation to reduce potential oxidative stress, preserve RNA quality, and increase cell viability.

scRNA-seq libraries were prepared using the Chromium Next GEM Single Cell 3’ v3.1 reagent kit (10x Genomics, Pleasanton, CA, USA). Cells were barcoded using a 10x Genomics Chromium Controller. Eleven polymerase chain reaction cycles were applied to generate cDNA, with thirteen cycles for final library generation. The final scRNA-seq libraries were sequenced with the Illumina NovaSeq X (Illumina, San Diego, CA, USA).

### Data analysis for whole-brain single-cell RNA sequencing

After receiving the raw FASTQ sequencing files, Cell Ranger’s (10x Genomics) count function was used to align sequencing reads to the GRCm38 *Mus musculus* reference genome. Count matrices were imported to R using the Seurat (v5.1.0) package’s Read10X function, and Seurat objects were created with sample-specific metadata.^139^ Cells were filtered based on quality control (QC) metrics (nCount_RNA > 800, nFeature_RNA > 200, mitochondrial percentage < 10%), data normalized with NormalizeData, and variable features identified with FindVariableFeatures. The data were scaled with ScaleData and dimensionality reduction was performed with RunPCA and RunUMAP. To remove doublets, DoubletFinder (v2.0.4) was used, with an expected doublet rate of 0.8% per 1,000 cells.^140^

To integrate data from multiple samples and minimize batch effects, batch correction was performed using the Harmony (v1.2.1) package with the “sample” variable as the integration factor.^141^ This approach preserves the underlying biological variation in gene expression within each cell type across treatment conditions and assumes that treatment condition does not drastically alter broad cell type identity (EC, astrocyte, mural cell, or fibroblast), while still allowing for large differences in subcluster composition due to the downstream analysis. Next, UMAP visualization and clustering on the Harmony-corrected embeddings (RunUMAP, FindNeighbors with dims = 1:50, FindClusters with resolution = 0.2) was performed. Clusters were annotated for cell types using canonical marker genes from PanglaoDB and CellMarker 2.0.^142,143^ BBB-relevant cell types were identified by expression of the following genes: *Cldn5*, *Flt1*, and *Cd93* for ECs; *Gfap*, *Aqp4*, and *Sox9* for astrocytes; *Rgs5*, *Pdgfrb*, and *Kcnj8* for mural cells; *Col1a1*, *Col1a2*, and *Lum* for fibroblasts. Marker genes for all other cell types can be found in Figure S4. For clearer visualization for the UMAP plot in Figure 3(a), the data were subset to include only BBB-relevant clusters (ECs, astrocytes, mural cells, and fibroblasts) and re-embedded using PCA (50 dimensions). The colorblind-friendly ggpubfigs (v0.0.1) palette was used for visualization of BBB-relevant cell types and some of the other cell types in Figure 3 and Figure S4.^144^

Subclustering analyses were then performed on ECs and astrocytes (RunPCA, FindNeighbors, FindClusters with resolution = 0.1 for ECs, 0.05 for astrocytes) to identify distinct transcriptional states within these populations. FindMarkers with the Model-based Analysis of Single-cell Transcriptomics (MAST) test was used to identify subcluster marker genes, comparing each individual cluster against all other cells within the same main cell type.^145^ The following parameters were used: latent variables of sex, mitochondrial percentage, and ribosomal percentage, a minimum expression threshold of 0.1 (expressed in at least 10% of cells), *p*_*adjusted*_ < 0.05, and log_2_FC ≥0.585 (at least a 50% difference in average expression compared to other clusters). These subclusters were named according to their marker genes (see Results). To assess subcluster enrichment between conditions, logistic regression was performed, controlling for sex.

For differential expression analysis between MTerm and MPreterm conditions for both overall cell types and subclusters, FindMarkers was used with the MAST test, with sex, mitochondrial percentage, and ribosomal percentage set as latent variables. Genes with adjusted *p*-value < 0.05 and |log_2_FC| ≥ 0.3785 (at least a 30% difference in average expression between conditions) were considered to be significantly differentially expressed. A list of all genes with a GO annotation of “synaptic signaling” (GO:0099536) were retrieved from the Jackson Laboratory Mouse Genome Informatics database to identify which DEGs were implicated in synaptic signaling.^146–148^

Gene set enrichment analysis was performed on the expression data, comparing between MTerm and MPreterm conditions. As the input for GSEA, the complete FindMarkers output (not only the DEGs) was used, creating ranked gene lists based on the log_2_FC values for each cell type and subtype. Gene symbols were converted to ENTREZ IDs using the org.Mm.eg.db (v3.19.1) mouse genome database package and gseGO from the clusterProfiler (v4.12.6) package was run with the following parameters: ont = “BP” (Biological Process), minGSSize = 10, and maxGSSize = 500.^149,150^ This allowed for the identification of enriched biological pathways and processes that were upregulated in either MTerm or MPreterm conditions, as indicated by positive or negative normalized enrichment scores, respectively, with significant pathways defined by *p*_*adjusted*_ < 0.05 (Benjamini–Hochberg procedure).

### Immunofluorescence

Mouse brains were freshly collected at two weeks postnatal and embedded in optimal cutting temperature compound before freezing. An 8 µm thick section from each mouse brain was fixed with ice-cold methanol for 20 min at −20°C. Samples underwent permeabilization with 0.2% Triton-X in PBS (PBST) for 15 min, followed by incubation in blocking solution (5% goat serum in PBST) for 1 h at room temperature. Brain sections were then incubated overnight with 50 μL of primary antibody solution at 4°C. After four PBST washes of 10 min each, sections were incubated with fluorophore-conjugated secondary antibodies for 1 h at room temperature. Primary antibodies used were rat anti-CD31 (cat#NB600–1475, Novus Biologicals, Centennial, CO, USA) and mouse anti-Cadherin-2 (cat#ab98952, Abcam, Cambridge, UK). Secondary antibodies used were goat anti-rat IgG, Alexa Fluor 488 (cat#A-11006, Thermo Fisher Scientific, Waltham, MA, USA) and goat anti-mouse IgG, Alexa Fluor Plus 647 (cat#A32728, Thermo Fisher). Nuclei were counterstained using ProLong Gold Antifade Mountant with DNA Stain DAPI (cat#P36935, Thermo Fisher). A STELLARIS confocal microscope (Leica Microsystems, Wetzlar, Germany) was used to capture five to seven images from each mouse brain. Image processing and analysis were performed using ImageJ.^151^ The BBB was identified via expression of CD31. CD31+ polygonal areas were outlined (Figure S5) and used for comparisons of fluorescence in Figure 3.

### Data collection for untargeted metabolomics

The data collection approach for the metabolomics experiment was followed as previously described.^72^ Serum metabolites were extracted by adding 100% methanol containing heavy-labeled internal standards (stored at −80 °C) to serum samples at a 1:10 ratio (100 µL serum:1,000 µL solvent). Samples were vortexed for 1 min, incubated overnight at −20°C, then centrifuged at −10°C, 20,000 × g for 15 min. Following centrifugation, 100 µL of supernatant was dried in a Genevac EZ-2 Elite (ATS Scientific Products, Warminster, PA, USA). Samples were resuspended in 200 µL of a 50/50 water/methanol solution, mixed at 4°C, 1,000 rpm for 15 min in an Eppendorf (Hamburg, Germany) ThermoMixer C, and centrifuged at 4°C, 20,000 × g for 15 min, after which 100 µL of clarified supernatant was transferred to MS vials. Analysis was performed with a Thermo Fisher liquid chromatography system coupled to an Orbitrap IQ-X mass spectrometer (Thermo Fisher) in positive mode, injecting 2 µL of sample onto a Cortecs UPLC T3 column (1.2 µm, 2.1 × 100 mm) (Waters Corporation, Milford, MA, USA) at 30°C, using water with 0.1% formic acid (mobile phase A) and 95% acetonitrile with 0.1% formic acid (mobile phase B). The gradient (0% B for 1 min, linear ramp to 100% B over 10 min, hold 2 min, re-equilibrate 2 min) flowed at 0.35 mL/min. Electrospray ionization parameters included 3.5 kV spray voltage, 400°C vaporizer temperature, and 133–2000 m/z detection window. MS2 scans employed dynamic exclusion after three selections of the same precursor ion within 15 s (exclusion duration 10 s), with an isolation window of 0.7 m/z (without offset) and fixed normalized collision energy of 35%.

### Data analysis for untargeted metabolomics

The metabolomics data analysis pipeline was applied as previously described.^72^ Feature lists were generated from raw data using MZmine (v2.53), beginning with mass detection set to noise cutoff thresholds of 8.0 × 10^3^ for MS2 and 2.0 × 10^4^ for MS1 scans.^152^ Extracted ion chromatograms were built using the ADAP chromatogram builder (minimum group size: 5 scans; group intensity threshold: 2.0 × 10^4^; minimum highest intensity: 4.0 × 10^4^; m/z tolerance: 0.01 Da or 3 ppm). Chromatograms were deconvoluted using the ADAP wavelets algorithm (S/N threshold: 10; minimum feature height: 6 × 10^4^; coefficient/area threshold: 110; peak duration: 0–1 min; RT wavelet range: 0.01–0.10 min). Isotopic peaks were grouped (maximum charge: 3; representative isotope: lowest m/z; tolerance: 0.01 Da or 3 ppm m/z, 0.1 min retention time (RT)), and features across samples were aligned (join aligner: 0.01 Da or 5 ppm m/z, 0.1 min RT). Duplicate features were filtered out, and missing peaks were gap-filled (peak-finder algorithm; 10% intensity tolerance, 0.01 Da or 5 ppm m/z, 0.1 min RT). Feature lists were exported for Global Natural Products Social Molecular Networking (GNPS) analysis.^153^ Feature-Based Molecular Networking^154^ via GNPS was used to filter MS/MS data (precursor and fragment tolerances: 0.01 Da; cosine score cutoff: 0.7; ≥ 4 matching peaks; mutual top-10 node connections; maximum family size: 100). Analog searching was set at a precursor mass difference ≤100 Da with identical filtering for library matches. GNPS-derived feature lists were further filtered in Excel based on blanks and QC injections to eliminate low-quality data. Peak areas within samples were normalized against the average area of internal standards (D_4_-cholic acid, D_4_-taurocholic acid, D_4_-glycocholic acid, D_4_-deoxycholic acid, D_4_-chenodeoxycholic acid,^13^C_9_-tyrosine, ^13^C_9_-phenylalanine, and ^13^C_11_-tryptophan). Three QC injections, using pooled samples, were used to assess reproducibility by excluding features with coefficient of variation > 20%. This resulted in a final list of 1,571 features; after removing known contaminants and polyether polymers, 231 putatively identified features remained. MetaboAnalyst was utilized to perform statistical analysis and visualization for both full and putatively identified feature lists, removing only features with single or constant values, without additional filtering or normalization.^155^

### Data collection for shotgun metagenomic sequencing

Fecal samples were collected from mice at four weeks postnatal and preserved at −80°C. Genomic DNA extraction and shotgun metagenomic sequencing were performed at the Duchossois Family Institute (DFI) Microbiome Metagenomics Facility using the Illumina NextSeq platform. Paired-end libraries were created in batches of 60–72 samples, with insert sizes of approximately 350 bp per sample. High-throughput sequencing generated about seven to eight million paired-end reads per sample with 150-bp read lengths.

### Data analysis for shotgun metagenomic sequencing

The raw sequencing data were processed by first trimming adapters off from raw reads and performing quality assessment and control with Trimmomatic (v0.39).^156^ Human genomic sequences were identified and removed using KneadData (v0.7.10).^157^ Taxonomic classification of the resultant high-quality reads was performed using MetaPhlAn (v4.0.2), and relative abundances were tabulated.^158^ Host-removed sequencing reads were also processed using HUMAnN (v3.8), and pathway abundances were normalized to relative abundances using the built-in normalization option.^157^

### 16S rRNA sequencing of human and mouse gut microbiota

These 16S rRNA-seq protocols were followed as previously described.^69,72,159^ Fecal samples from the human infant donors were stored at −80°C and subsequently submitted for genomic DNA extraction and subsequent 16S rRNA-seq via the GS-FLX platform with XLR70 sequencing chemistry (Roche, Basel, Switzerland) at the Institute for Genomics and Systems Biology at Argonne National Laboratory.^160,161^ PCR primers targeting the V3–V4 regions of the 16S rRNA gene (nucleotide positions 338–802 in *Escherichia coli*) were used. These primers were constructed with 454 sequencing-compatible adapter sequences and unique 8-nucleotide barcode identifiers. The resulting sequence data were processed and merged with DADA2 in QIIME2 (v2019.10).^162,163^ Taxonomic assignment used the QIIME2 naïve Bayes classifier trained on the SILVA database (release 138).^164^

Mouse fecal samples were collected at two weeks postnatal, four weeks postnatal, and from dams. These samples were stored at −80°C. They were then submitted to the DFI Microbiome Metagenomics Facility for genomic DNA extraction before 16S rRNA-seq using the Illumina MiSeq. Sequencing reads were processed in R using DADA2 (v1.18.0) with minor modifications.^162^ Forward and reverse reads were truncated to 190 bp to remove low-quality tails, and chimeric sequences were discarded via DADA2’s consensus algorithm. Amplicon sequencing variants between 320 bp and 365 bp were retained as high-quality. Taxonomy was assigned with the RDP classifier (v2.13, training set 18/release 11.5) with 80 as the minimum bootstrap confidence score.^165^

### Statistical analyses

For both the fear conditioning and open field tests, the Shapiro-Wilk test was used to determine whether behavioral outcomes were normally distributed at *α* = 0.05 for both MTerm and MPreterm mice. If a given behavioral outcome was determined to be non-normally distributed for at least one of the treatment groups, then the data was log-transformed for hypothesis testing. If not, the data remained unchanged. Then, Welch’s *t*-test was performed. For the Morris water maze, a two-way ANOVA was performed with treatment and training day as predictors. The same statistical tests were performed to test for sex differences as were used to test for differences due to treatment but with Benjamini–Hochberg adjustment of the *p*-values for the fear conditioning and open field test readouts to account for the same outcome being queried twice (once in the MTerm comparison and once in the MPreterm comparison).

For determination of DEGs in scRNA-seq data, FindMarkers with MAST *p*-value adjustment was used.^139^ Gene set enrichment analysis was performed with gseGO, with the Benjamini–Hochberg procedure to adjust *p*-values.^150^ For comparison of subclusters, a logistic regression model was fit to the subclustering data to retrieve odds ratios. The corresponding *p*-values were calculated via the Wald test and Bonferroni-adjusted. Wilcoxon rank sum tests were used for comparison of fluorescence in immunostaining images.

In comparisons of brain regional volume and BBB permeability, the Shapiro–Wilk test was applied to assess whether measurements were normally distributed at *α* = 0.05 for both treatment groups. If, for a given brain region, the data were non-normally distributed for at least one of the MTerm or MPreterm groups, then the data were log-transformed for hypothesis testing. The data remained unchanged otherwise. Welch’s *t*-test was subsequently performed.

For the identification of microbial metabolites present at altered levels in MTerm and MPreterm mice, unpaired *t*-tests were used before adjustment by the Benjamini, Krieger, and Yekutieli procedure.^166^

In the analysis of shotgun metagenomic data, the Wilcoxon rank sum test with Benjamini–Hochberg adjustment was utilized to compare α-diversity and relative abundances of bacterial taxa and genomic pathways. For further comparison between MTerm and MPreterm gut microbiota, a Bray-Curtis dissimilarity matrix was computed using vegdist from the vegan package.^167^ PERMANOVA (adonis2) with 10^5^ permutations was used, with treatment as the independent variable.

For 16S rRNA-seq analysis, the modified Wilcoxon rank sum test for zero-inflated data^168^ was used for comparisons of relative abundance of bacterial taxa, and *p*-values were adjusted via the Benjamini–Hochberg procedure. To assess β-diversity, a Bray-Curtis dissimilarity matrix was constructed, and PERMANOVAs were performed with 10^5^ permutations, with either treatment or age as the independent variable.

## Supplementary Material

Supplemental Material

## Data Availability

scRNA-seq data have been deposited to the Gene Expression Omnibus (accession: GSE291278). Metabolomics data have been deposited to MassIVE (dataset ID: MSV000097292). Metagenomics data have been deposited to the Sequence Read Archive (BioProject ID: PRJNA1236379). Code for this study is available via Zenodo (DOI: 10.5281/zenodo.16897951).
