# Supplementary material for: Early-life gut microbiome maturity regulates blood–brain barrier and cognitive development
Source: Gut Microbes. 2025 Aug 31;17(1):2551879. doi: 10.1080/19490976.2025.2551879 (PMC12416178; doi:10.1080/19490976.2025.2551879)
Supplement: Supplemental Material [file KGMI_A_2551879_SM6616.pdf]

Supplemental Materials: *Early-life gut microbiome maturity regulates blood-brain barrier and cognitive development*

<u>Included</u>
Figures S1 to S5
Tables S3 & S10
Access Instructions for Tables S1 to S11

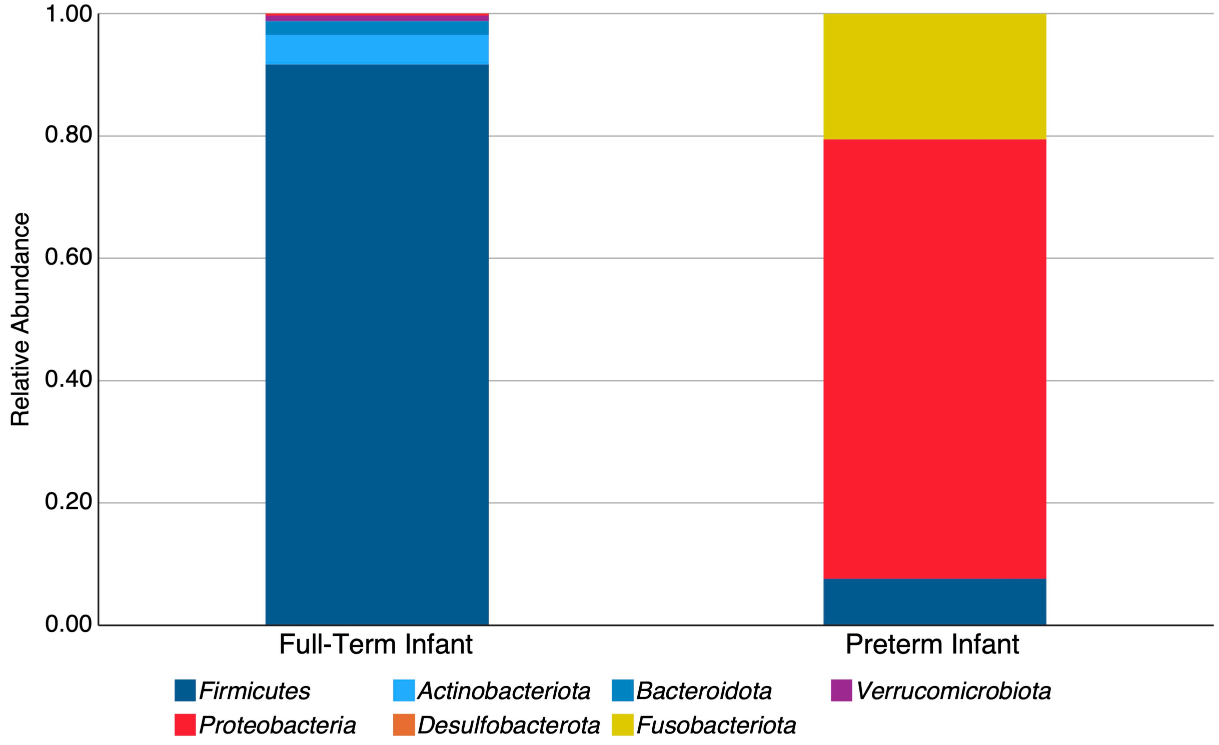

Figure S1. Phylum-level relative abundances of gut bacteria from human donors. Full-term infant: GA = 39 weeks. Preterm infant: GA = 24 weeks.

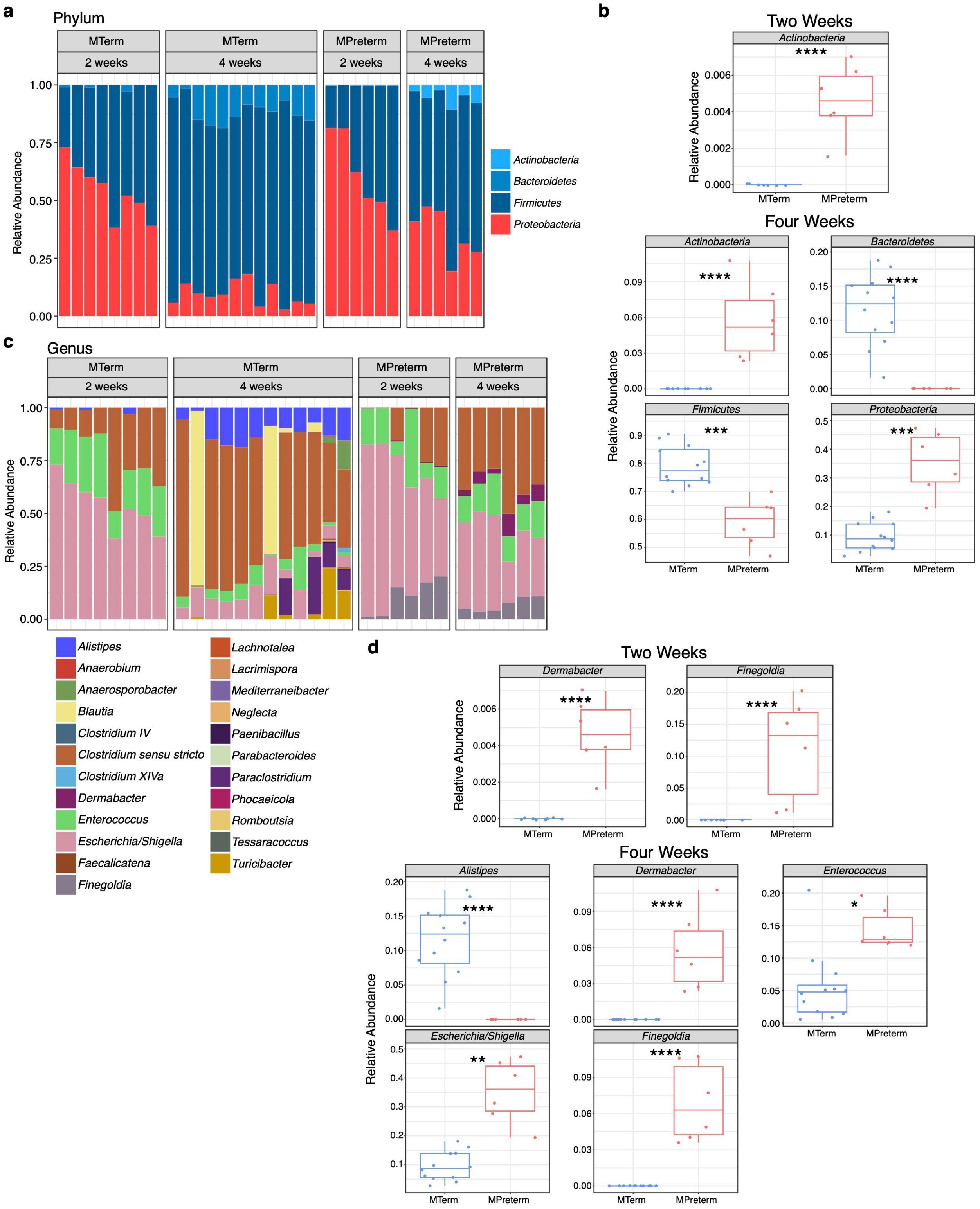

Figure S2. Relative abundances of MTerm and MPreterm gut microbiota at two and four weeks of age, as determined via 16S rRNA-seq. * $p_{adjusted} < 0.05$, *** $p_{adjusted} < 0.01$, *** $p_{adjusted} < 0.001$, **** $p_{adjusted} < 0.0001$, Benjamini-Hochberg-adjusted modified Wilcoxon rank sum test for zero-inflated data. (a) Phylum-level relative abundances. (b) Phyla present at significantly different levels in MTerm and MPreterm microbiota at two (top) or four (bottom) weeks of age. (c) Genus-level relative abundances. (d) Genera present at significantly different levels in MTerm and MPreterm microbiota at two (top) or four (bottom) weeks of age.

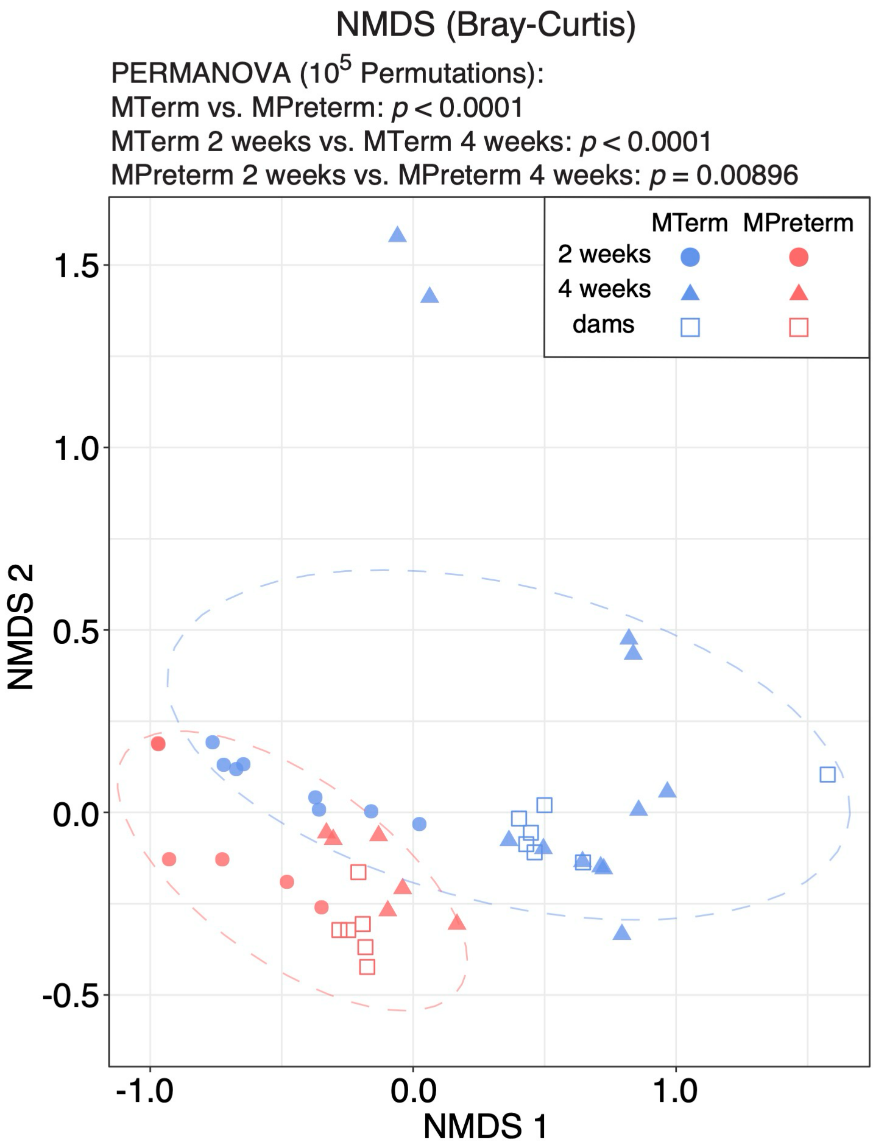

Figure S3. Non-metric multidimensional scaling (NMDS) of MTerm and MPreterm microbiomes using 16S rRNA-seq data.

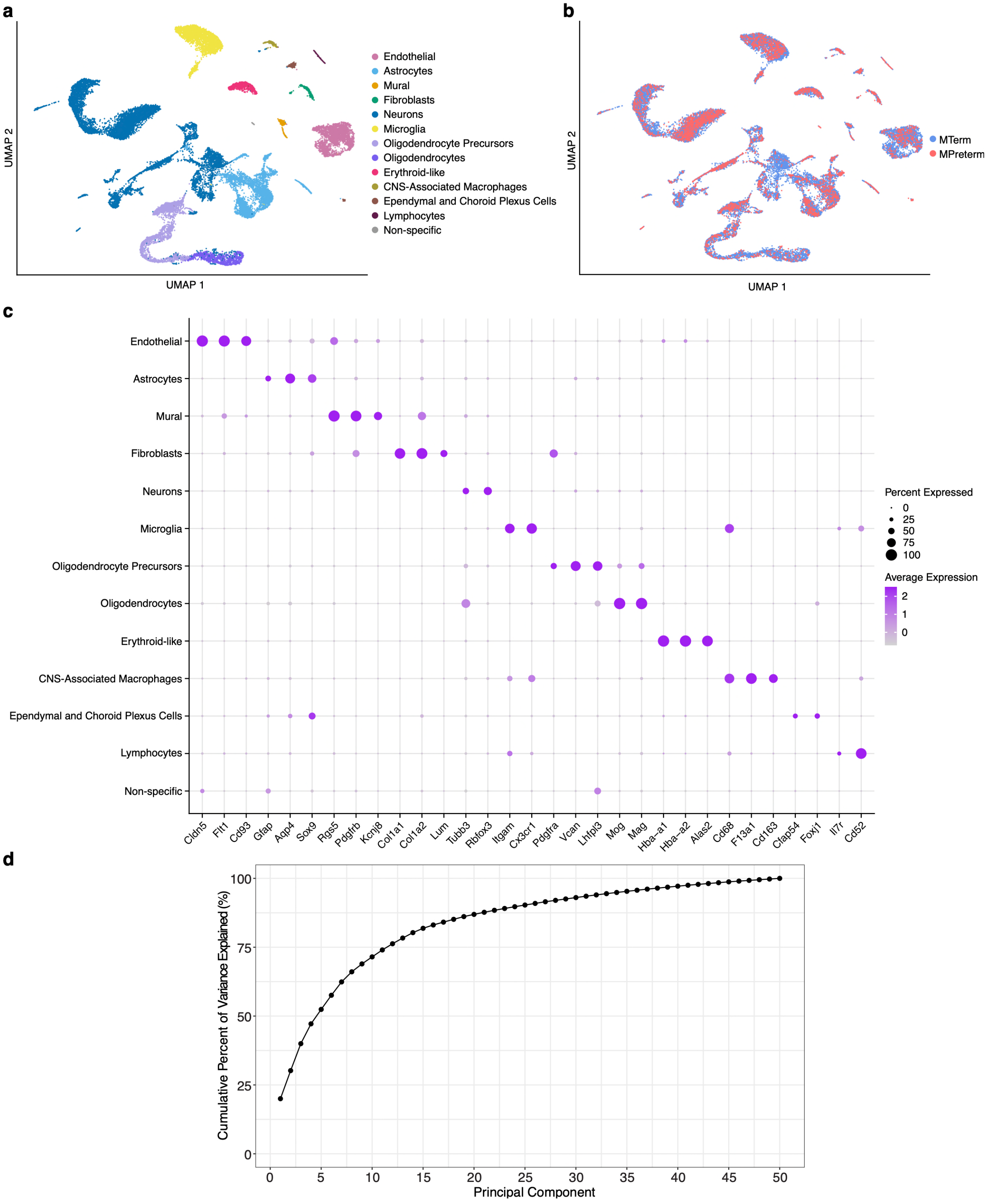

Figure S4. Additional analysis of scRNA-seq data. (a-b) UMAP visualizations of clustered scRNA-seq data colored by (a) cell type or (b) treatment. (c) Dot plot showing relevant marker genes for each cell type. Dot size represents the percentage of cells within a sub-cluster expressing a given gene, and dot color represents the average expression of that gene across the sub-cluster relative to other sub-clusters. (d) Plot showing the cumulative variance of total gene expression explained by each successive Harmony-integrated principal component.

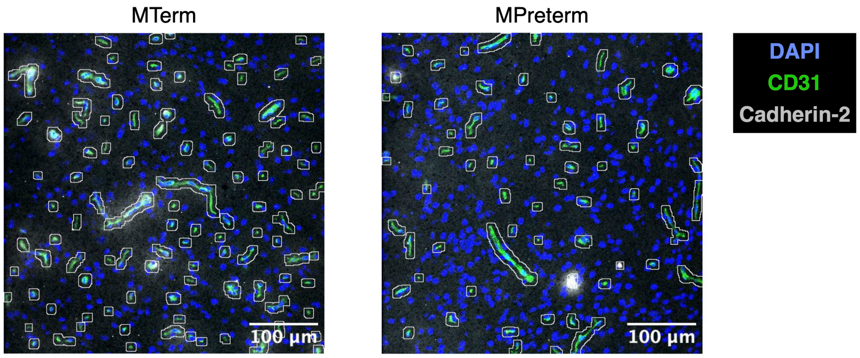

Figure S5. Representative images displaying polygonal selections of CD31+ regions used to identify BBB location. CD31 (green) and Cadherin-2 (gray) expression at the BBB (as seen in Figure 3) were determined based on these selections in ImageJ. Nuclei are stained with DAPI (blue). Scale bar = 100 µm. Left: MTerm brain. Right: MPreterm brain.

Two Weeks					
	MTerm Mean	MTerm SEM	MPreterm Mean	MPreterm SEM	Significance
Body Weight (g)	8.134	0.36	8.488	0.46	n.s.
**Brain Volume (mm3)**					
Total	380.1	4.98	390.2	0.1	n.s.
Cerebellum	45.17	0.51	45.95	0.02	n.s.
Corpus callosum	9.163	0.13	9.47	0.02	n.s.
Cortex	122.4	1.59	125.4	0.09	n.s.
Hippocampus	28.36	0.41	29.13	0.04	n.s.
Hypothalamus	13.18	0.17	13.48	0.02	n.s.
Medulla	26.32	0.38	27.11	0.03	n.s.
Midbrain	27.24	0.42	28.22	0.04	n.s.
Olfactory	23.5	0.26	23.98	0.06	n.s.
Pallidum	9.546	0.24	10.15	0.02	$p = 0.0421$
Pons	15.42	0.3	16.06	0.04	n.s.
Striatum	35.53	0.53	36.59	0.04	n.s.
Thalamus	14.82	0.14	14.92	0.01	n.s.
Ventricles	2.2	0.03	2.264	0.01	n.s.
White matter	7.236	0.11	7.423	0.03	n.s.
**Three Weeks**					
Body Weight (g)	9.846	0.71	10.38	0.74	n.s.
**Brain Volume (mm3)**					
Total	398.2	5.58	386.7	5.51	n.s.
Cerebellum	47.18	0.82	45.96	0.65	n.s.
Corpus callosum	9.609	0.13	9.324	0.13	n.s.
Cortex	128.1	1.92	124.5	1.78	n.s.
Hippocampus	29.82	0.39	28.86	0.43	n.s.
Hypothalamus	13.77	0.24	13.42	0.19	n.s.
Medulla	27.42	0.26	26.48	0.42	n.s.
Midbrain	28.67	0.35	27.84	0.4	n.s.
Olfactory	24.64	0.44	23.99	0.33	n.s.
Pallidum	10.1	0.06	9.739	0.19	n.s.
Pons	16.23	0.15	15.69	0.25	n.s.
Striatum	37.35	0.45	36.19	0.53	n.s.
Thalamus	15.43	0.33	15.04	0.22	n.s.
Ventricles	2.303	0.03	2.25	0.03	n.s.
White matter	7.58	0.08	7.347	0.11	n.s.

Table S3. Comparison of volumes of fourteen different brain regions between MTerm and MPreterm mice at two and three weeks of age.

Experiment	Treatment Group	Age (weeks)	Number of Female Mice	Number of Male Mice	Total *n*	Number of Litters Used
Fear Conditioning	MTerm	4	9	4	13	2
	MPreterm	4	11	11	22	4
Morris Water Maze	MTerm	4	5	4	9	2
	MPreterm	4	7	9	16	3
Open Field	MTerm	4	8	4	12	2
	MPreterm	4	11	11	22	4
MRI (Regional Volume)	MTerm	2	5	3	8	4
	MPreterm	2	4	4	8	5
	MTerm	3	2	5	7	3
	MPreterm	3	5	4	9	5
MRI (DCE)	MTerm	2	5	3	8	4
	MPreterm	2	4	2	6	5
	MTerm	3	2	6	6	3
	MPreterm	3	5	4	9	5
scRNA-seq	MTerm	2	3	1	4	2
	MPreterm	2	2	2	4	1
Immunofluorescence	MTerm	2	2	4	6	4
	MPreterm	2	1	2	3	3
Untargeted Metabolomics	MTerm	4	5	1	6	2
	MPreterm	4	4	2	6	3
Shotgun Metagenomics	MTerm	4	6	5	11	5
	MPreterm	4	3	3	6	3
16S rRNA-seq	MTerm	2	3	5	8	3
	MPreterm	2	3	3	6	3
	MTerm	4	9	3	12	3
	MPreterm	4	3	3	6	3
	MTerm	dams	6	0	6	N/A
	MPreterm	dams	6	0	6	N/A

Table S10. Sample sizes and breakdown by treatment, age, and sex for all experiments.

All supplemental tables are available as Excel files via Figshare (DOI: 10.6084/m9.figshare.29176220).

Legends for Tables S1 to S11 are as follows:

Table S1. Relative abundances of gut bacteria at phylum and genus level in human donors.

Table S2. Statistical analyses for behavioral assays, including tests for sex differences. Tab 1: Fear conditioning. Tab 2: Open field test. Tab 3: Morris water maze.

Table S3. Comparison of volumes of fourteen different brain regions between MTerm and MPreterm mice at two and three weeks of age.

Table S4. Significant DEGs between MTerm and MPreterm mice in BBB-relevant cell types. Tab 1: Endothelial cells. Tab 2: Astrocytes. Tab 3: Mural cells. Tab 4: Fibroblasts.

Table S5. GSEA results for comparisons between MTerm and MPreterm BBB-relevant cell types. Tab 1: Endothelial cells. Tab 2: Astrocytes. Tab 3: Mural cells. Tab 4: Fibroblasts.

Table S6. Synaptic signaling-annotated genes (GO:0099536) differentially expressed in MTerm and MPreterm endothelial cells and astrocytes. Tab 1: Endothelial cells. Tab 2: Astrocytes.

Table S7. GSEA results for comparisons between MTerm and MPreterm endothelial and astrocyte sub-clusters**.** Tab 1: AEAs. Tab 2: ARAs. Tab 3: IMAs. Tab 4: SCAs. Tab 5: SSAs. Tab 6: AVECs. Tab 7: BR-CECs. Tab 8: VM-CECs.

Table S8. Levels of all metabolites captured from MTerm and MPreterm serum.

Table S9. Metagenomic pathways present at significantly different levels in MTerm and MPreterm mice.

Table S10. Sample sizes and breakdown by treatment, age, and sex for all experiments.

Table S11. Number of litters and number of mice used from each litter for each experiment.
